# Genome-Wide Collation of the *Plasmodium falciparum* WDR Protein Superfamily Reveals Malarial Parasite-Specific Features

**DOI:** 10.1371/journal.pone.0128507

**Published:** 2015-06-04

**Authors:** Priyanka Chahar, Manjeri Kaushik, Sarvajeet Singh Gill, Surendra Kumar Gakhar, Natrajan Gopalan, Manish Datt, Amit Sharma, Ritu Gill

**Affiliations:** 1 Centre for Biotechnology, Maharshi Dayanand University, Rohtak, Haryana, India; 2 DRDO-BU Centre for Life Sciences, Bharathiar University Campus, Coimbatore, Tamil Nadu, India; 3 Structural and Computational Biology Group, International Centre for Genetic Engineering and Biotechnology, Aruna Asaf Ali Marg, New Delhi, India; Philipps-University Marburg, GERMANY

## Abstract

Despite a significant drop in malaria deaths during the past decade, malaria continues to be one of the biggest health problems around the globe. WD40 repeats (WDRs) containing proteins comprise one of the largest and functionally diverse protein superfamily in eukaryotes, acting as scaffolds for assembling large protein complexes. In the present study, we report an extensive *in silico* analysis of the WDR gene family in human malaria parasite *Plasmodium falciparum*. Our genome-wide identification has revealed 80 putative WDR genes in *P*. *falciparum* (*Pf*WDRs). Five distinct domain compositions were discovered in *Plasmodium* as compared to the human host. Notably, 31 *Pf*WDRs were annotated/re-annotated on the basis of their orthologs in other species. Interestingly, most *Pf*WDRs were larger as compared to their human homologs highlighting the presence of parasite-specific insertions. Fifteen *Pf*WDRs appeared specific to the *Plasmodium* with no assigned orthologs. Expression profiling of *Pf*WDRs revealed a mixture of linear and nonlinear relationships between transcriptome and proteome, and only nine *Pf*WDRs were found to be stage-specific. Homology modeling identified conservation of major binding sites in *Pf*CAF-1 and *Pf*RACK. Protein-protein interaction network analyses suggested that *Pf*WDRs are highly connected proteins with ~1928 potential interactions, supporting their role as hubs in cellular networks. The present study highlights the roles and relevance of the WDR family in *P*. *falciparum*, and identifies unique features that lay a foundation for further experimental dissection of *Pf*WDRs.

## Introduction

WD40 repeats (WDRs) or WD40 domain proteins comprise one of the largest and functionally diverse protein families in eukaryotes from yeast to human. The WD40 domain is among the top ten most abundant domains in eukaryotic genomes [1]. Though rare, these proteins are present in some prokaryotes e.g. *Thermomonospora curvata* and *Cyanobacterium synechocystis* [1,2]. The WDR family is defined by a sequence repeat of ~40 amino acids that characteristically begins with a glycine-histidine (GH) pair at N-terminus and ends with a tryptophan-aspartic acid pair (WD) at C-terminus [2]. Typically the WD40 proteins are composed of several tandem repeats of WD40 motifs [3]. The WD40 repeat motif is comprised of a four-stranded antiparallel β-sheet (a-b-c-d) and displays only limited amino acid sequence conservation [1,2,4].

Despite significant level of sequence diversity, the WD40 repeats invariably fold into a β-propeller conformation with number of blades corresponding to the number of repeating units present [4–7]. Each WD40 β-propeller blade contains the first three strands of one repeat unit and the last strand of the previous repeat (d-a-b-c). Thus, the four strands of WD40 repeat belong to two different WD40 blades. This sharing of strand between two blades and hydrophobic interactions between the blades results in stabilization of the β-propeller structure [2,8].

The members of WDR family have been characterized to play important role in multiple cellular processes, such as RNA processing, signal transduction, vesicular trafficking, regulation of cell division, apoptosis, ubiquitination/protein degradation, chromatin assembly, remodeling and transcriptional regulation [4,9–11]. The underlying common feature of all the members of WDR family is their coordination of multiprotein complex assemblies. These repeats provide a stable platform or scaffold on which large protein or protein-DNA complexes can assemble. As determined by the available WD40 domain complex structures, the WDR proteins have three distinct surfaces (top, bottom and circumference) that can be exploited for interaction with other proteins [1,11]. The ability of this domain to interact simultaneously with a number of proteins through multiple interaction surfaces makes them important players in the cellular interaction networks. The WD40 domain is one of the top interacting domains in eukaryotic genomes. It has been identified to participate in more interaction pairs than any other domain in yeast [1]. The versatile functions of WDR family seem to be the outcome of participation in multiple protein-protein interactions [1] and fusion with additional domains i.e. multidomain architecture [3,12,13].

Peculiar features of the WDR gene family are- i) low sequence conservation although high evolutionary conservation; ii) co-occurrence of the WD40 domain with other domains; iii) interaction with multiple proteins to form large complexes and; iv) the functional diversity. Despite of the global reduction in malaria mortality by 42% from 2000 to 2012, malaria continues to be a major public health problem threatening 3.4 billion people across 97 countries and causing deaths of ~627,000 people in 2012 [14]. The WDR gene family remains uncharacterized in *Plasmodium falciparum* (the most virulent species of human malaria) except for a few individual protein studies of this family e.g. *Pf*Sec31, *Pf*Sec13 and *Pf*RACK [15–17]. In the present study, a comprehensive genome-wide analysis of the WDR family in *P*. *falciparum* has been performed in order to understand the significance, functionality and evolutionary relationships. Additionally, we have investigated the expression patterns of *Pf*WDRs through different developmental stages of *Plasmodium* based on the available transcriptome and proteome datasets. Homology modeling enabled three-dimensional (3D) structure prediction and comparisons to human orthologs. We have also investigated protein-protein interactions (PPIs) networks for *Pf*WDRs, and highlighted their potential significance in parasite biology.

## Results and Discussion

### Identification and listing of *Pf*WDR genes in *P*. *falciparum*

**Fig 1:**
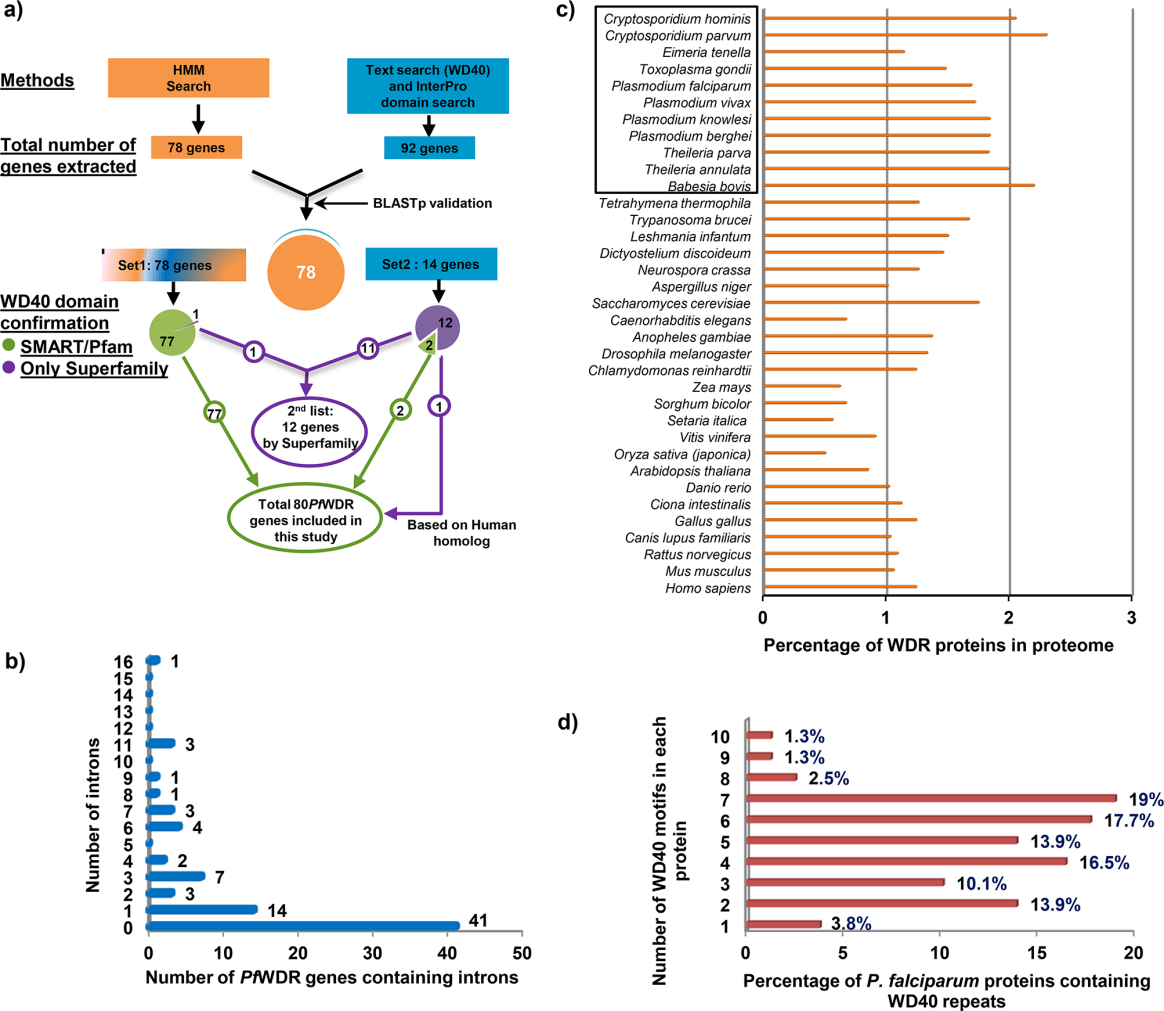
Extraction and characterization of the *Pf*WDRs. a) Schematic representation of the approaches employed for the identification of *Pf*WDR genes. b) Graphical representation of the occurrence of introns by number in *Pf*WDR genes. c) Predicted percentage of the proteome of eukaryotic organisms devoted for the WDR proteins. Apicomplexans are boxed. d) Distribution of the WD40 motifs by number in *Pf*WDRs.

To identify genes of the WDR family in *P*. *falciparum*; we used two approaches (Fig 1A). First, HMM search was performed using WD40 family seed downloaded from Pfam which resulted in the identification of 78 genes. Second, a keyword search ‘WD40’ and the InterPro domain search (IPR017986) against PlasmoDB resulted in the extraction of 92 putative *Pf*WDR genes. Further, BLASTp analysis with the yeast and human WD40 sequences as queries was also done to validate the above results. A careful analysis and union of both the lists resulted in the identification of 92 putative *Pf*WDR genes. The intersection of both the lists contained 78 genes and was taken as the first set (Set1) of putative *Pf*WDR genes (Fig 1A). Second set (Set2) composed of 14 putative *Pf*WDR genes after subtracting first set from the total 92 putative *Pf*WDR genes.

Subsequently, both the sets of putative *Pf*WDR proteins were subjected to Pfam and SMART analysis to confirm the presence of WDRs. These databases confirmed the presence of WD40 repeat motifs in 79 proteins; 77 from Set 1 and 2 from Set 2 resulting in the first list of 79 putative *Pf*WDR genes (Table A in S1 Table). In the remaining genes, WDRs were recognized by Superfamily database only and were kept in the second list of putative *Pf*WDR genes (Table B in S1 Table) and eliminated from further analysis. However, one gene based on its human homolog named as WDR8 was extracted from second list and included in first list resulting in the final list of 80 putative *Pf*WDR genes (Table A in S1 Table). Presence of large number of WDR genes in *P*. *falciparum* is in accordance with other eukaryotic organisms, where WD40 domain has been shown to be one of the most abundant domains [1].

Further, data on CDS length, protein length, molecular weight, number of introns, and chromosome location for the putative *Pf*WDR genes was extracted from PlasmoDB (S1 Table). Out of the 80 *Pf*WDR genes, 39 genes (48.8%) contained introns that roughly corroborates with the reported distribution of introns in *P*. *falciparum* genome i.e. 53.9% of *P*. *falciparum* genes possess introns [18]. The number of introns in *Pf*WDRs varied from 1 to 16 (Fig 1B) with PF3D7_1037800 (a cytoskeletal regulatory protein) having maximum number of introns. Length and molecular weight of the *Pf*WDRs varied from 323 aa and 35.7 kDa (PF3D7_0526300, PF3D7_0826700) to 4405 aa and 526.7 kDa (PF3D7_1410300). There were large variations in the pI values, ranging from 4.37 to 9.8. These variations in pI and molecular weight of *Pf*WDRs are comparable to the WDR proteins known in other organisms e.g. *Arabidopsis thaliana* [3], *Oryza sativa* [12], *Setaria italica* (foxtail millet) [13] and *Homo sapiens* [19] etc.

### Intergenomic analysis

To perform comparative analysis of the abundance of WDR proteins in different eukaryotes, we extracted WDR proteins from several organisms as reported in various studies or as per SMART database or general text and InterPro domain search (IPR017986). There were 237 WDR proteins in *A*. *thaliana* [3], 200 in *O*. *sativa* [12], 225 in *S*. *italica* [13] and 267 in *H*. *sapiens* [19]. Fig 1C shows the percentage of WDR proteins in the proteomes of different eukaryotes (S2 Table). In comparison to plants and metazoans, apicomplexans possessed relatively higher percentage of the WDRs as per proteome size. This may be because of the participation of WDRs in basic cellular and metabolic processes and less complexity of the apicomplexans as compared to the plants and metazoans resulting in higher percentage of WDRs as per their proteome size.

### Number of WDR motifs

WD40 proteins are characterized by the presence of multiple tandem repeats generally varying from 4 to 8 [2]. However, proteins with minimum 2 and maximum 16 WDRs have also been reported [3,10,20]. In our analysis, a total of 354 WDR motifs were recognized by SMART among 80 *Pf*WDRs which range from 1–10 repeats/protein (Fig 1D). Approximately 70% of all the *Pf*WDR proteins have 4–8 WD40 repeats. Pfam database recognized less number of WD40 proteins as well as number of repeats as compared to SMART (Table A in S1 Table) thus seems to be more stringent in the identification of members of this family.

### Generation of sequence logo

**Fig 2:**
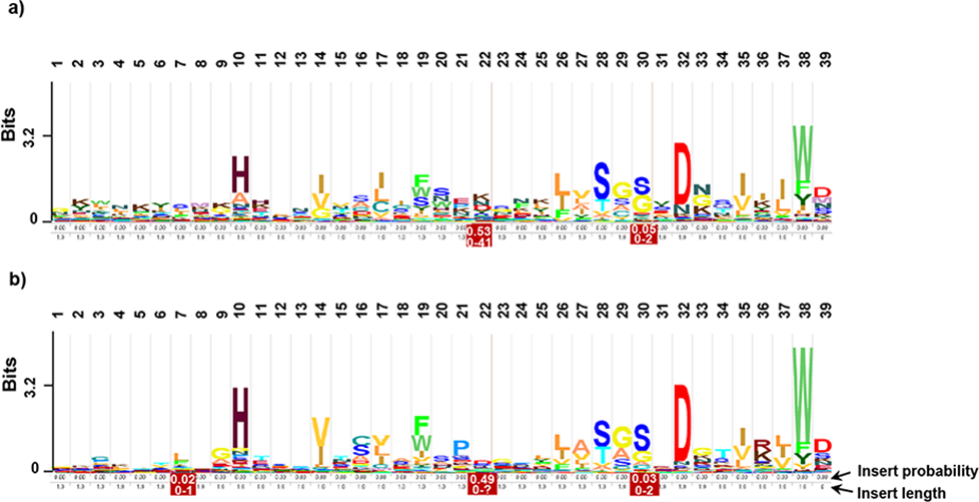
Sequence logos for the WD40 motif. a) The *Pf*WDRs HMM logo based on alignment of all the identified WDRs in *P*. *falciparum*. b) The Pfam WD40 family HMM logo drawn from alignment of Pfam WD40 seed sequences.

To explore the characteristics and extent of sequence conservation of the WD40 repeats in *P*. *falciparum*, their sequence logo was generated and subsequently compared with the HMM logo of Pfam WD40 family. As per the definition of WDR superfamily, the characteristic features of the family were presence of GH dipeptide at N-terminus ad WD dipeptide at C-terminus. Nevertheless, as clear in the generated logos (Fig 2), even these positions were not conserved thus making identification of the family members and all the repeats in a protein difficult by sequence comparison methods. Further, as per the Pfam logo, the most conserved positions in the WD40 superfamily were H_10_, D_32_ and W_38_. These positions showed significant conservation in the *Pf*WDRs logo but less than Pfam HMM logo, revealing more variations within the *Pf*WDR superfamily at these positions. The most conserved residue in the *Pf*WDRs was D_32_. HMM seed of high confidence *Pf*WDR sequences was built and has been provided as File B in S1 File. This can be used to identify WDR proteins in other organisms.

### Domain architecture analysis of *Pf*WDR proteins

**Fig 3:**
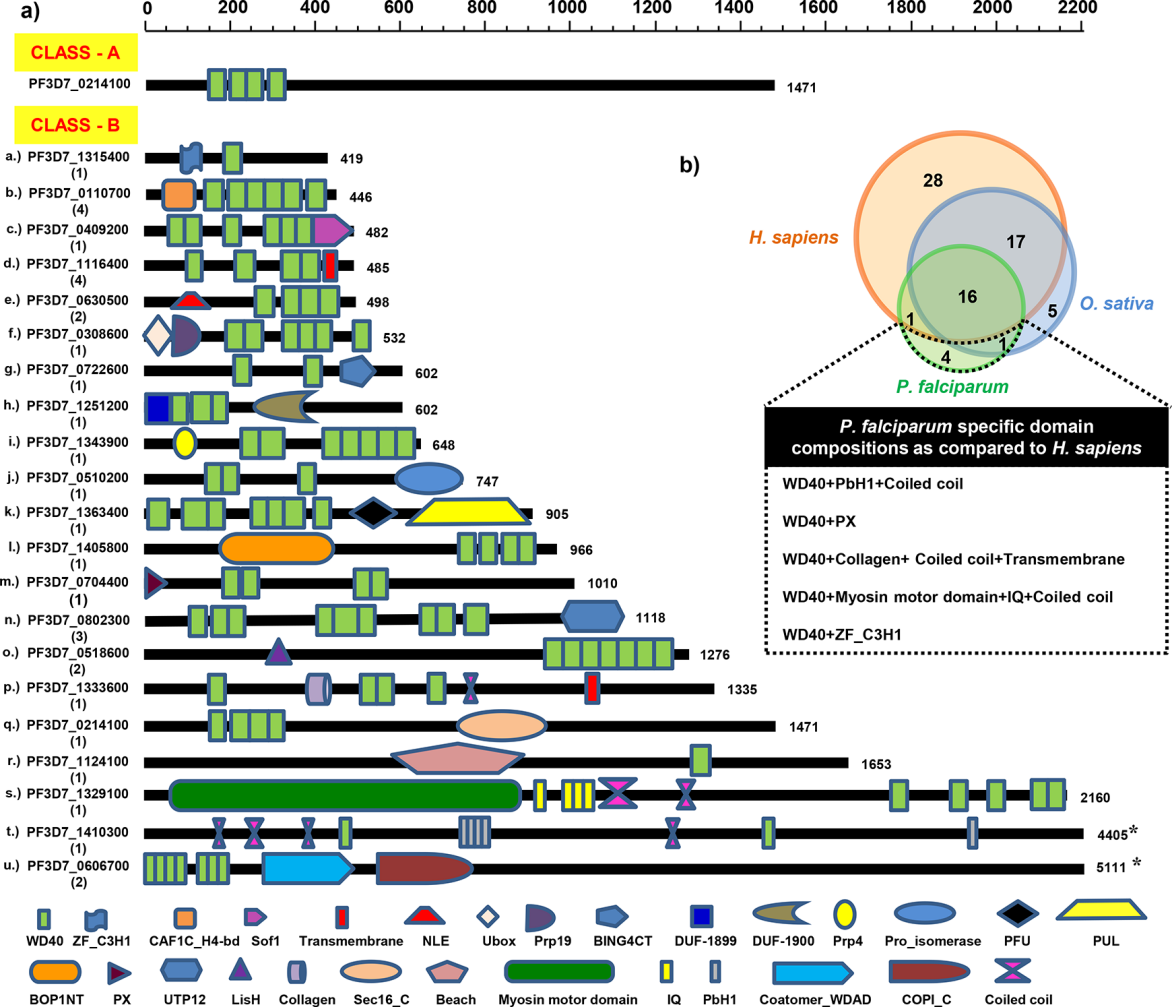
Domain organization of the *Pf*WDRs a) Domain organization of the representative *Pf*WDRs from each class and subclass based on the identification of WD40 and other additional domains by SMART, Pfam and InterPro. Subclass name and gene ID for each protein are given on the left and the number of members of each subclass is given in parenthesis. Domain positions are scaled according to the protein length bar given at the top except gene IDs marked with asterisk (٭) b) Venn diagram depicting the number of shared and specific domain combinations of *P*. *falciparum*, *H*. *sapiens* and *O*. *sativa* WDR proteins. Inset table enlists the *P*. *falciparum* specific domain compositions as compared to the human host.

Domain composition analysis for the 80 *Pf*WDR proteins as per SMART and Pfam databases revealed that 48 proteins had only WD40 domain (Class A), whereas, 32 proteins showed WD40 domain in combination with other functional domains (Class B). Class B was further divided in 21 subclasses (**a-u**) based on the type of additional domains present (Fig 3A). Importantly, five domain compositions of *Pf*WDRs were found to be specific to the parasite as compared to their human host (Fig 3B). Subclass-**a** has one member (PF3D7_1315400) having a combination of WD40 domain with ZF_C3H1 (CCCH type zinc finger). This domain combination has not been identified in human although present in some plants, alveolates and fungi. Subclass-**h** has one member (PF3D7_1251200) exhibiting two unique domains of unknown functions (DUF1899 and DUF1900) interspaced by three WDRs, which is the characteristic feature of coronin gene family [21]. However, a C-terminal variable coiled coil domain responsible for oligomerization (one of the characteristic feature of short coronins) was absent in PF3D7_1251200. A medley of PX with WD40 was identified unique to *Plasmodium* as compared to its human host (subclass **m**- PF3D7_0704400). The PX-WD40 fusion is conserved in *Plasmodium* species, apicomplexans and restricted to only alveolates.

PF3D7_1333600 having a combination of collagen, transmembrane domain and coiled coils with three WD40 motifs was kept in subclass-**p**. This domain architecture is unique to *Plasmodium* although combination of WD40+collagen is present in other organisms. Another subclass-**s** (PF3D7_1329100) featured the presence of N-terminal myosin motor domain (head domain), multiple IQ motifs (neck domain) and coiled coils, which are usually associated with the myosin heavy chains [22]. The combination of WD40 with the N-terminal coiled coils in variable tail domain of myosin heavy chains is exclusively present in the apicomplexans. Subclass-**t** has one member (PF3D7_1410300) with PbH1 (parallel beta helix repeats) domain and coiled coils in addition to the WD40 domain. This domain composition is absent in human.

**Fig 4:**
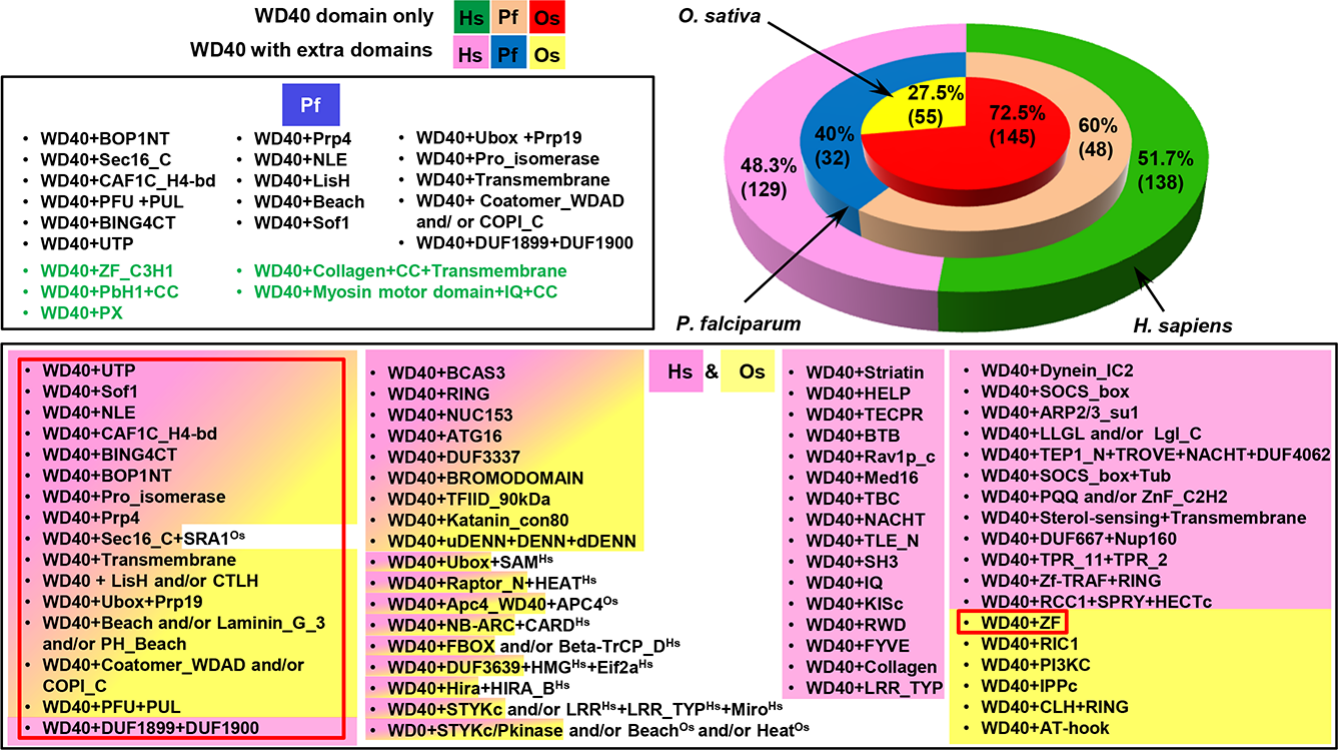
Comparison of percentage occurrence of the WDR proteins containing WD40 domain alone or in combination with additional domains in *H*. *sapiens* (*Hs*), *P*. *falciparum* (*Pf*) and *O*. *sativa* (*Os*) [12] shown by stacked pie diagram. *P*. *falciparum* specific domain compositions as compared to *H*. *sapiens* are highlighted in the green text. Domain compositions in pink, yellow and pink-yellow shades are of human, rice and shared human-rice, respectively as per SMART database. Superscripts ‘Hs’ and ‘Os’ represent domains specifically present in human and rice. Domain compositions of human and rice shared with *P*. *falciparum* are enclosed in the red box.

A comparison of the domain organization of WDRs in *P*. *falciparum*, *H*. *sapiens* (as per SMART database) and *O*. *sativa* [12] is illustrated in Fig 4. Our results showed that ~40% and ~48% WDRs in *P*. *falciparum* and *H*. *sapiens* have multidomain combinations, respectively. However, in *O*. *sativa* ~27% WDRs were reported to be associated with additional domains [12]. A fusion with additional domains may contribute to the functional diversity of WDRs.

### Functional insights based on orthologs of *Pf*WDRs

To investigate the putative functions of *Pf*WDRs, we analyzed the cellular function of each listed member by exploring gene annotations in PlasmoDB and annotations of orthologs at UniProt aided by domain composition analysis and published articles. Ascribing putative roles to *P*. *falciparum* proteins based on homology is challenging due to lack of sufficient sequence similarity to the characterized genes in other organisms. This is because of the extremely AT-rich *P*. *falciparum* genome that results in an unusual amino acid composition, presence of large insertions and low complexity regions [18,23].

Orthologs for the *Pf*WDRs were predicted using BLAST/PSI-BLAST search as mentioned in the methods and compiled in S3 and S4 Tables. Importantly, while assigning orthologs we considered retention of key domains, comparable protein sizes and annotation of the reference proteins. Notably, it was not always the first hit in BLAST that has been assigned as a homolog. Sometimes, no significant hit was observed directly in *H*. *sapiens*; however, homolog was assigned through BLAST searches against other organisms. For example, *Hs*WDR12 showed a weak hit against PF3D7_0630500 in BLAST (S3 Table), whereas, its yeast ortholog Ytm1 is a significant first hit (79% sequence coverage, 24% identity and 6e-18).

We were able to assign human orthologs to 61 out of 80 *Pf*WDRs (S3 Table) though assignment of orthologs based on sequence similarity searches was poorer. Notably, most of the *Pf*WDRs (90%) were of longer length as compared to their human orthologs highlighting the presence of species-specific insertions in *Plasmodium*. Further, the length of three *Pf*WDRs was almost twice of their assigned orthologs (S3 Table) that may be because of the fusion of two proteins or large *Pf* specific insertions e.g. *Pf*Sec13 is a combination between Sec13 and Nup145C of yeast [16]. No human ortholog could be assigned to PF3D7_1333600 that has been named as U3 snoRNA-associated small subunit rRNA processing protein. Moreover, this annotation was not conserved amongst *Plasmodium* species. Brehelin et al. [24] annotated the protein based on guilt-by-association principle and assigned its yeast ortholog Utp4. Accordingly, we assigned its human ortholog Utp4; however, there was variation in length and domain structure of PF3D7_1333600 and its assigned human ortholog (S3 Table).

Orthologs for all the *Pf*WDRs were also searched in apicomplexans *Babesia bovis* and *Toxoplasma gondii*. Except for the 17 *Pf*WDRs, we could identify orthologs for all the others either in *B*. *bovis*/ *T*. *gondii* or both (S4 Table). Further, for the 19 *Pf*WDRs (with no human orthologs) orthologs were searched in other *Plasmodium* species, apicomplexans, alveolates, yeast, *Drosophila* and *Arabidopsis*. This analyses revealed 1 apicomplexan specific, 2 alveolates specific, 1 alveolata and plants specific, 14 *Plasmodium* genus specific and 1 *P*. *falciparum* and *Plasmodium reichenowi* specific WDR proteins (Table B in S4 Table).

**Fig 5:**
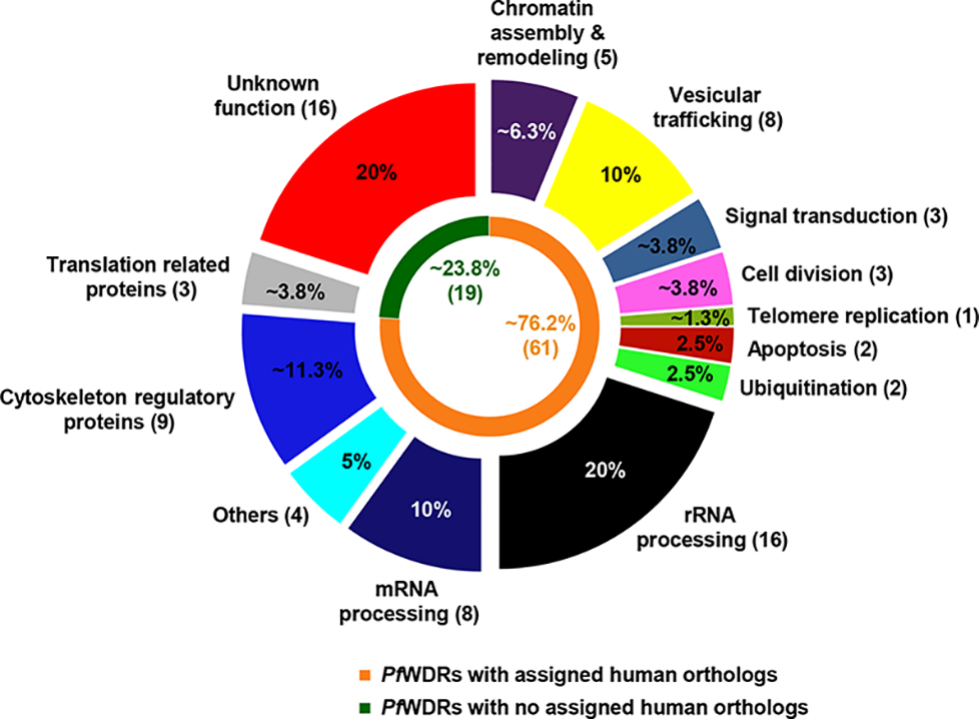
Pie chart representing the functional classification of *Pf*WDRs and the number of assigned human orthologs (S3 Table). The *Pf*WDRs are categorized into 13 functional classes. Number of proteins assigned to each class (given in parenthesis) and their percent to the total number of identified *Pf*WDRs are indicated. Inner pie chart represents percent and number of *Pf*WDRs with assigned human orthologs.

By homology based inter-species annotation transfer, we were able to annotate/reannotate 31 *P*. *falciparum* proteins which were previously unannotated or misannotated or having less refined annotation (Table 1). Further, *Pf*WDRs were manually categorized into 13 different generic groups (Fig 5, S3 Table). A total of 16 *Pf*WDRs, for which no function could be predicted, were kept in unknown function category. The largest fraction of *Pf*WDRs was predicted to be involved in the RNA processing. Functional diversity of the *Pf*WDRs is in accordance with other eukaryotes, where WDRs were shown to be involved in a wide range of cellular functions [4,10].

**Table 1:** List of *Pf*WDRs with suggested annotation/refined reannotation based on orthologs.

Gene ID	Annotation at PlasmoDB	Human orthologs	Suggested reannotation
PF3D7_1243800	Microtubule associated katanin, putative	NP_079498.2-WD repeat containing protein 82 (YKL018-COMPASS component SWD2)	*Pf*SWD2 or *Pf*WDR82
PF3D7_1347000	G-beta repeat protein, putative	NP_612467.1-WD repeat containing protein 92/Monad	*Pf*WDR92 or Monad
PF3D7_0518600	WD-repeat protein, putative	NP_079436.3-WD repeat containing protein 26 (YCL039W-Glucose-induced degradation protein 7 (GID7))	*Pf*WDR26
PF3D7_1467200	Conserved Plasmodium protein, unknown function	NP_060551.1-Telomerase Cajal body protein 1 (TCAB1)/WD repeat containing protein 79	*Pf*TCAB1 or *Pf*WDR79
PF3D7_0608000	Conserved Plasmodium protein, unknown function	NP_620133.1-Diphthamide biosynthesis protein 7 (DPH7)/WD repeat containing protein 85 (YBR246W-Diphthamide biosynthesis protein 7)	*Pf*DPH7 or *Pf*WDR85
PF3D7_1118800	Conserved Plasmodium protein, unknown function	NP_006400.2-Actin-related protein 2/3 complex subunit 1A (YBR234C-Actin-related protein 2/3 complex subunit 1)	Actin-related protein 2/3 complex subunit 1A
PF3D7_0510800	Conserved Plasmodium protein, unknown function	NP_078808.3-Sperm associated antigen 16 protein/Pf20 protein homolog	Pf20 protein homolog
PF3D7_1348700	Conserved Plasmodium protein, unknown function	NP_659491.4-Cilia and flagella associated protein 52 (CFAP52)/WD repeat-containing protein 16	CFAP52 or *Pf*WDR16
PF3D7_1406500	Conserved Plasmodium protein, unknown function	NP_689711.1-WD repeat containing protein 65 (Trypanosome homolog: Tb927.8.4870- DIGIT)	*Pf*DIGIT
PF3D7_1033500	WD-repeat protein, putative	NP_060504.1-WD repeat containing protein 70 (*C*. *elegans* homolog: O16519-Gastrulation defective protein 1 (GAD-1))	*Pf*WDR70 or *Pf*GAD-1
PF3D7_1105200	Conserved Plasmodium protein, unknown function	NP_060288.2-WD repeat containing protein WRAP73/WDR8	*Pf*WDR8
PF3D7_1221600	Conserved Plasmodium protein, unknown function	NP_003301.1-Tumor-suppressing STF cDNA 1 protein (TSSC1)	*Pf*TSSC1
PF3D7_1428400	Probable protein, unknown function	NP_055838.2-WD and tetratricopeptide repeats protein 1 (WDTC1)	*Pf*WDTC1
PF3D7_0409200	40S ribosomal processing protein, putative	NP_056235.3-DDB1 and CUL4 associated factor 13 (DCAF13) (YLL011W*-nucleolar RNA-associated protein SOF1)	*Pf*DCAF13 or Protein SOF1
PF3D7_1237600	rRNA processing WD-repeat protein, putative	NP_008993.1-Periodic tryptophan protein 1 homolog (PWP1) (YLR196W*-Periodic tryptophan protein 1)	*Pf*PWP1
PF3D7_1405800	Large subunit rRNA processing protein, putative	NP_056016.1-Ribosome biogenesis protein BOP1 (YMR049C*-Ribosome biogenesis protein ERB1)	*Pf*BOP1 or *Pf*ERB1
PF3D7_0816000	Nucleolar preribosomal assembly protein, putative	NP_113673.2-Glutamate-rich WD repeat-containing protein1 (GRWD1) (YMR131C*-Ribosome assembly protein RRB1)	*Pf*RRB1 or *Pf*GRWD1
PF3D7_1146000	Nucleolar preribosomal assembly protein, putative	NP_060566.2-Notchless protein homolog 1 (NLE1) (YCR072C-Ribosome assembly protein 4 (RSA4))	*Pf*NLE1 or *Pf*RSA4
PF3D7_0802300	rRNA processing WD-repeat protein, putative	NP_005040.2-Periodic tryptophan protein 2 homolog (PWP2) (YCR057C*-Periodic tryptophan protein 2 (UTP1))	*Pf*UTP1
PF3D7_1333600	U3 snoRNA-associated small subunit rRNA processing associated protein, putative	NP_116219.1-Cirhin/UTP4 (YDR324C^**#**^-U3 small nucleolar RNA-associated protein 4)	*Pf*UTP4^**#**^
PF3D7_0722600	Nucleolar rRNA processing protein, putative	NP_005443.3-WD repeat containing protein 46 (YER082C*-U3 small nucleolar RNA-associated protein 7 (UTP7))	*Pf*UTP7
PF3D7_1448000	U3 snoRNA-associated small subunit rRNA processing protein, putative	NP_006775.1-WD repeat containing protein 3 (YLR129W*-U3 small nucleolar RNA-associated protein 12 (UTP12)/DIP2)	*Pf*UTP12
PF3D7_1013100	U3 snoRNA-associated small subunit rRNA processing protein, putative	NP_006444.2-Transducin beta-like protein 3 (TBL3) (YLR222C*-U3 small nucleolar RNA-associated protein 13 (UTP13))	*Pf*UTP13
PF3D7_1352200	Conserved Plasmodium protein, unknown function	NP_115551.2-U3 small nucleolar RNA-associated protein 15 homolog (UTP15)	*Pf*UTP15
PF3D7_1357700	U3 snoRNA-associated small subunit rRNA processing protein, putative	NP_644810.1-WD repeat containing protein 36 (YLR409C*-U3 small nucleolar RNA-associated protein 21 (UTP21))	*Pf*UTP21
PF3D7_1226700	Conserved Plasmodium protein, unknown function	NP_004695.1-RNA U3 small nucleolar interacting protein 2/RRP9 homolog (YPR137W-Ribosomal RNA-processing protein 9 (RRP9))	*Pf*RRP9
PF3D7_0630500	Microtubule-associated protein ytm1 homologue, putative	NP_060726.3-Ribosome biogenesis protein WDR12 (YOR272W-Ribosome biogenesis protein YTM1)	Ribosome biogenesis protein YTM1 or *Pf*WDR12
PF3D7_0801500	Conserved Plasmodium protein, unknown function	NP_079170.2-Nucleolar protein10 (NOL10) (YGR145W-Ribosome biogenesis protein ENP2)	*Pf*NOL10 or Ribosome biogenesis protein ENP2 homolog
PF3D7_1220100	Pre-mRNA splicing factor, putative	NP_056975.1-Pre-mRNA-processing factor 17 (PRP17) (YDR364C-Pre-mRNA-processing factor 17)	*Pf*PRP17
PF3D7_0302000	Golgi organization and biogenesis factor, putative	NP_002660.1-Pleiotropic regulator 1 (PLRG1) (YPL151C-Pre-mRNA-splicing factor PRP46)	*Pf*PRP46 Pre-mRNA-splicing factor
PF3D7_1241100	Conserved Plasmodium protein, unknown function	NP_060853.3-Pre-mRNA 3' end processing protein WDR33 (YNL317W-Polyadenylation factor subunit 2 (PFS2))	*Pf*WDR33 or *Pf*PFS2
PF3D7_0905600	Conserved Plasmodium protein, unknown function	NP_653269.3-WD repeat containing protein 66 isoform 1	*Pf*WDR66
PF3D7_1329100	Myosin C (MyoC)	-	Myosin F^**^**^

*Saccharomyces cerevisiae*/*Trypanosoma brucei*/*Caenorhabditis elegans* orthologs are mentioned (in brackets in column 3) where annotations are based on these.

‘*’ indicates orthologs/reannotation also suggested by Brehelin et al. [24] or Ochoa et al. [68].

‘#’ and ‘^’ indicate orthologs/annotations given by Brehelin et al. [24] and Foth et al. [22] respectively.

### Subcellular targeting of *Pf*WDRs

**Fig 6:**
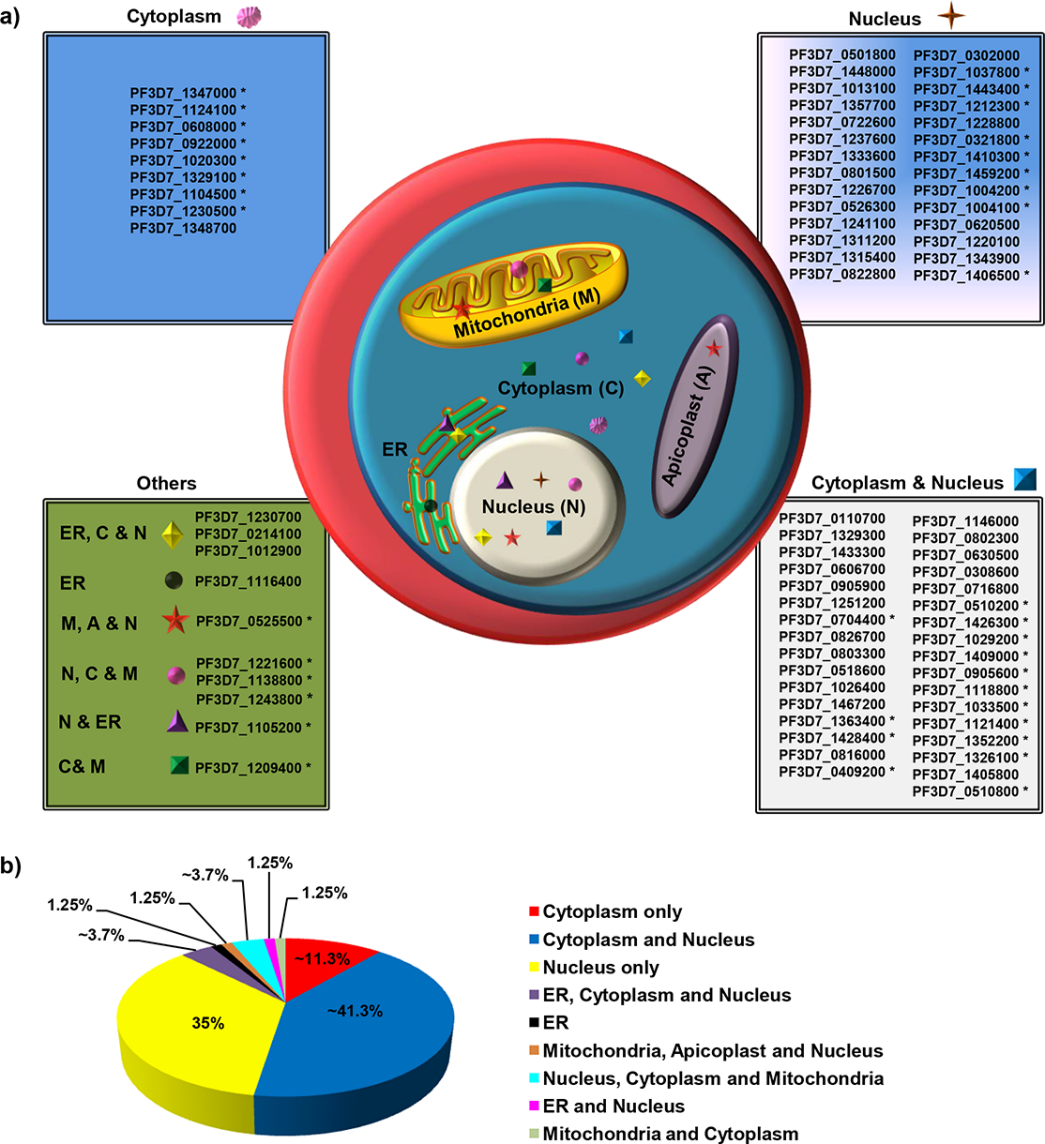
Predicted subcellular localization of the *Pf*WDRs (S5 Table). a) A schematic representation of the subcellular localization of *Pf*WDR proteins based on online programs and literature review. Abbreviations are as follows: C, cytoplasm; N, nucleus; ER, endoplasmic reticulum; M, mitochondria; A, apicoplast. b) Percentage predicted distribution of *Pf*WDRs in different organelles within the protozoan parasite. Localization of gene IDs marked with asterisk was predicted *in silico*.

Functional diversity of the *Pf*WDRs necessitates their localization in various cellular compartments. Accordingly, *Pf*WDRs were predicted to localize in various subcellular compartments viz. cytosol, nucleolus, nucleus, endoplasmic reticulum (ER), mitochondria and apicoplast (Fig 6). Subcellular localization was predicted on the basis of available experimental evidence/literature review either for *Pf*WDR or its ortholog and where no supporting literature was available, prediction was based on bioinformatics tools as mentioned in the methods. Most *Pf*WDRs (86.3%) were predicted to reside in the nucleus, which is in agreement with their major role in RNA processing, chromatin assembly and remodeling. Out of these, ~35% *Pf*WDRs were exclusively present in the nucleus. Interestingly, subcellular targeting of most (15/17) of the *Plasmodium* specific conserved proteins with no assigned ortholog has been predicted to be nuclear by various programs.

Apicoplast and mitochondria are the two organelles with extra-chromosomal DNA in *Plasmodium*. None of the *Pf*WDRs were predicted to localize in the apicoplast. However, PF3D7_0525500 was predicted to possess apicoplast targeting transit peptide but the protein lacks signal peptide. Five *Pf*WDRs possessed a potential mitochondrion-targeting signal sequence as defined by Mitoprot. Further, no *Pf*WDR was found to have PEXEL motif. Importantly, 52.5% *Pf*WDRs were predicted to localize in more than one compartment, thus arguing for their multiple roles. Accuracy of various bioinformatics tools in predicting subcellular localization of *Pf*WDRs seems to be low in comparison to localization inferred from direct experimentation either for the ortholog proteins or *Pf*WDR proteins (S5 Table). For example, in comparison to experimental data (either for *Pf*WDRs or their orthologs), Euk-mPLoc and NetNES mispredicted localization (mislocalized or not able to predict main organelle) of ~61% and ~48% *Pf*WDRs, respectively. Low precision of the subcellular localization prediction for *Pf*WDRs by different programs suggests the presence of divergent signal sequences in *Plasmodium* as compared to other eukaryotes.

### Expression profiles of *Pf*WDRs at mRNA level

To examine the expression profiling of *Pf*WDRs, we took advantage of the extensive transcriptome and proteome data available at PlasmoDB. First, we analyzed the transcriptome data from Llinas/Derisi et al. [25] and Le Roch/Winzeler et al. [26]. Derisi group represented the transcriptome profiling of intraerythrocytic developmental cycle (IDC) at one hour time resolution with 53 time points for *P*. *falciparum* 3D7 dataset. While, Winzeler group examined nine different stages: seven periodic erythrocytic asexual stage time points i.e. early and late rings (ER & LR), trophozoites (ET & LT), schizonts (ES & LS) and merozoites (M) with IDC synchronized by two different methods; sorbitol and temperature, the sexual stage gametocytes (G) and mosquito salivary gland sporozoites (Sp).

**Fig 7:**
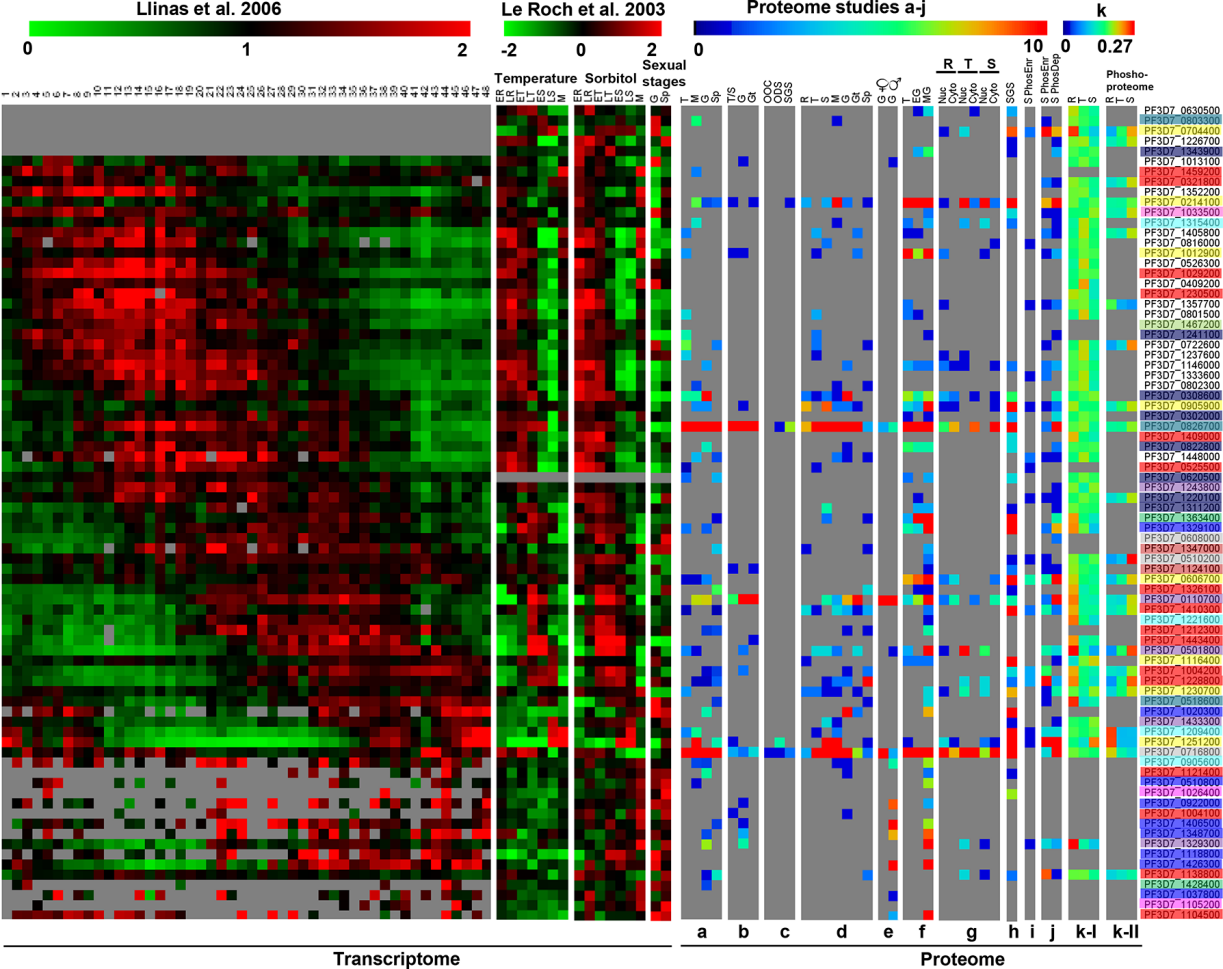
Expression patterns of the *Pf*WDR genes during life cycle of the parasite. A *Pf*WDRs phaseogram from microarray data of Llinas/Derisi et al. [25] was generated covering IDC (1–48h) and compared with Le Roch/Winzeler et al. [26] data from two independently synchronized *P*. *falciparum* 3D7 cultures i.e temperature and sorbitol covering IDC stages (R,T,S,M) as well as G and Sp. Colorimetric representation used for heat maps of transcriptome data is green-red (green, low expression; black, medium expression; red, high expression). Heat map panels at the right side with blue-red colour scale (blue, low expression; red, high expression) represent comparison of proteome and phosphoproteome data obtained from (**a**) Florens et al. [27], (**b** & **c**) Lasonder et al. [28,29], (**d**) Le Roch et al. [30], (**e**) Khan et al. [31], (**f**) Silvestrini et al. [32], (**g**) Oehring et al. [33], (**h**) Linder et al. [34], (**i**) Solyakov et al. [35], (**j**) Treeck et al. [36] and (**k**-I & **k**-II) Pease et al. [37]. Column to the right indicates PlasmoDB gene IDs of *Pf*WDRs coloured according to the functional classification (see Fig 5). Different life cycle stages are abbreviated as: ER and LR, early and late rings; ET and LT, early and late trophozoites; ES and LS, early and late schizonts; M, merozoites; G, gametocytes; Sp, sporozoites; Gt, gamete; EG, early gametocyte; MG, mature gametocyte; OOC, oocyst; ODS, oocyst derived sporozoites; SGS, salivary gland sporozoites; phosEnr, phospho-enriched; phosDep, phospho-depleted; Nuc, nuclear; and cyto, cytoplasmic. Grey colour represents absence of detection.

A phaseogram of the *Pf*WDRs was constructed by arranging Derisi IDC transcriptome dataset according to the phase of gene expression and compared with accordingly arranged Winzeler’s transcriptome heatmap produced from log_2_ ratio of RMA expression value to average RMA value for all the time points for a gene (Fig 7). Overall there was good agreement between the two data sets with respect to *Pf*WDRs. As per both transcriptome studies, most of the *Pf*WDRs’ transcripts were upregulated in R and T stage with only few *Pf*WDRs upregulated in S stage (Fig 7). As per Winzeler dataset, some *Pf*WDRs were found to be preferentially expressed in G and/or Sp and/or M and low expression level throughout IDC stages R, T and S e.g. most of cytoskeleton regulatory genes (PF3D7_0510800, PF3D7_0922000, PF3D7_1406500, PF3D7_1348700, PF3D7_1426300, PF3D7_1037800), two cell division genes (PF3D7_1026400, PF3D7_1105200) and some conserved proteins unknown function (PF3D7_1104500, PF3D7_1121400). Transcripts of these genes were either not detected in Derisi dataset or detected in only few time points probably because of low transcript abundance throughout IDC.

To highlight the differential transcript abundance of the *Pf*WDRs, we reorganized Winzeler dataset in four clusters from low to high transcript abundance (S1 Fig). Of note, functionally co-related *Pf*WDRs e.g. those involved in rRNA processing and mRNA processing were found to be co-expressed in-phase (Fig 7) in order to ensure the presence of all the products to carry out a particular function.

### Proteome profiles of *Pf*WDRs and comparison with transcriptome data

Next, we addressed the correlation between the transcriptome profiling and actual protein expression of *Pf*WDRs by compiling the proteome data from 11 different studies available at PlasmoDB and labelled as **a** to **k** covering 7 different developmental stages of complex *Plasmodium* life cycle (Fig 7) as follows: **(a)** Florens et al. [27], **(b&c)** Lasonder et al. [28,29], **(d)** Le Roch et al. [30], **(e)** Khan et al. [31], **(f)** Silvestrini et al. [32], **(g)** Oehring et al. [33], **(h)** Linder et al. [34], **(i)** Solyakov et al. [35], **(j)** Treeck et al. [36] and **(k)** Pease et al. [37]. A total of 77 *Pf*WDRs were identified by all the different proteome studies compiled here, which is almost equal to the total number of identified transcripts. The most comprehensive proteome analysis of *Pf*WDRs was provided by study **(k)** identifying 58 *Pf*WDRs in asexual stages (R,T,S).

Most of the *Pf*WDRs classified as cytoskeleton regulatory proteins were G and Sp specific as per their functional classification (Fig 7). These *Pf*WDRs showed low transcript expression profile throughout IDC with little or significant upregulation either in S and/or M and/or G and/or Sp (Fig 7, S1 Fig) indicating well coordination in transcriptome and proteome Myosin F (PF3D7_1329100) was observed to have good mRNA expression throughout IDC, G and Sp. Accordingly, its protein was found in R, T, S, G, Sp. PF3D7_1026400-cell division cycle protein 20 (CDC 20) homolog has been shown to be highly upregulated in Sp at mRNA level and its protein has been detected in only Sp by report **(h)**. However, Guttery et al. [38] showed that *P*. *berghei* CDC20 was highly expressed at mRNA and protein level in male G and also present throughout the life cycle of malarial parasite with no upregulation in Sp. PF3D7_1104500 showed upregulation of mRNA in G by Winzeler dataset and accordingly its protein was detected in male G by reports **(e)** & **(f)**. The gene remained unannotated, however, BLAST analysis showed *Hs*POC1 as a noteworthy hit (S3 Table) though coiled coil domain (characteristic feature of POCs) seems to be absent in *P*. *falciparum*. The POC1 proteins are the constituents of centriole having a major role in formation of cilia and flagella. Nevertheless, a detailed phylogenetic analysis of the Poc1 proteins showed absence of these proteins in *Plasmodium* [39].

The *Pf*WDRs involved in rRNA and mRNA processing in accordance with their function were found to be present throughout IDC- R,T,S and some were also detected in G, Sp and M by various proteome studies. Transcripts of all rRNA processing genes were upregulated from R to T stage and the relative abundance of their proteins was higher in T except for PF3D7_0630500 as shown by study **(k)**. As per study **(g)** out of 15 predicted rRNA processing *Pf*WDRs, only three (PF3D7_1357700, PF3D7_1237600, PF3D7_1146000) were detected to be nuclear. This could be due to either less efficient methods of extraction of nuclear proteome or low abundance of protein to be detected by mass spectrometry.

Despite overall coordination, significant contradictions between different proteome studies have also been noted. PF3D7_0525500 protein was detected in T by studies **a**, **d** and in S by study **i**. However, other studies (**b**, **g** and **k**) including analysis of both these stages did not detect any peptide belonging to this protein. Similarly, mRNA level of PF3D7_0608000 was upregulated in T and Sp. However, its protein was detected in S only by study **j,** whereas, none of the other proteome study detected its presence, pointing towards lack of coordination between various proteome studies as well as nonlinear relationship between transcript and protein.

Only a few *Pf*WDRs were stage specific i.e. confined to one or two stages (S2 Fig). Nine *Pf*WDRs were present in only one stage and six *Pf*WDRs were restricted to two stages. Thus, except few *Pf*WDRs, all are resident of multiple life cycle stages highlighting their role in basic cellular and molecular processes throughout the life cycle of *Plasmodium*. *Pf*WDRs restricted to single stage were either cytoskeleton regulatory proteins or conserved proteins with unknown function that may be playing parasite stage specific roles in cell division, invasion, motility etc. Out of 9 *Pf*WDRs confined to a single stage, 7 *Pf*WDRs transcripts were identified to be in coordination with proteome (S2 Fig). No correlation was seen between transcripts and proteome abundance in the *Pf*WDRs restricted to two stages except PF3D7_1121400. In addition, some highly abundant *Pf*WDRs transcripts showed high protein expression (PF3D7_0826700, PF3D7_0716800, PF3D7_0214100) (Fig 7, S1 Fig). However, it is not true for all and disagreement is quite common between level of transcript and protein abundance (PF3D7_1029200, PF3D7_1352200, PF3D7_1230700, PF3D7_0110700).

Thus, here we present detailed expression analyses of the *Pf*WDRs revealing a mixture of linear and nonlinear relationships between the transcriptome and proteome. Nonlinear relationships highlight presence of regulatory mechanisms at transcript as well as protein level. Literature revealed only a handful of studies so far that draw relationships between global or gene family transcriptome and proteome in *Plasmodium* [40–43]. Our efforts to illustrate the degree of correlation between the *Pf*WDRs transcriptome and proteome are significant. The study highlights importance of future experimental efforts that aim at dissecting regulation mechanisms in *Plasmodium*.

### Genomic localization of *Pf*WDRs

**Fig 8:**
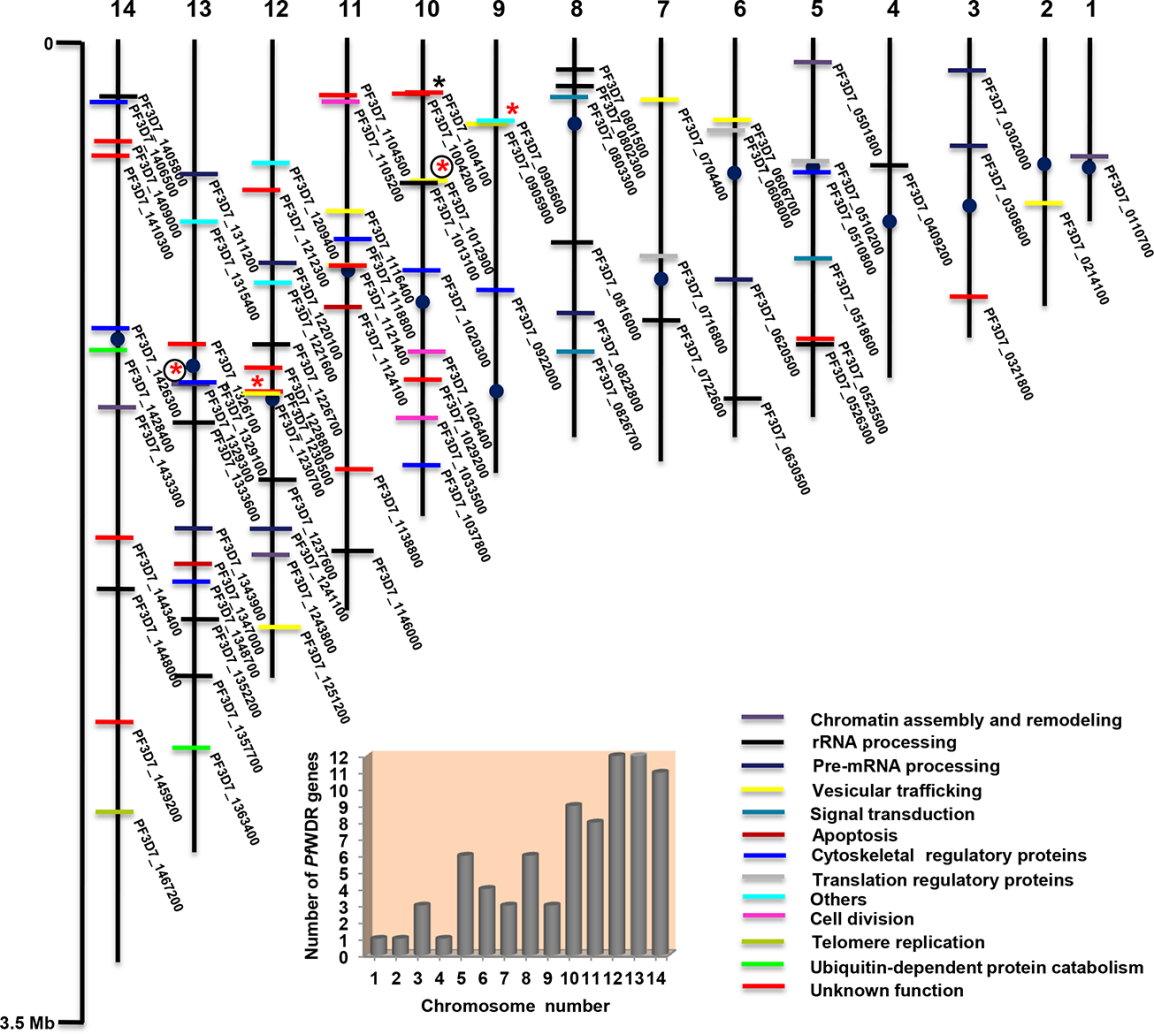
Physical mapping of *Pf*WDR genes depicting their genomic localization onto 14 chromosomes of *P*. *falciparum*. Positions of centromeres are represented by filled circles on the chromosomes (vertical bars). Integers at the top of each bar indicate chromosome number. Grouped genes, adjacent genes and genes leaving one or two gene positions in between; are highlighted with black and red asterisks, respectively. Further, asterisks for clusters having co-expressed genes are encircled. Genes on chromosomes are colour coded as per their functional classification (see Fig 5). The scale on the left is in megabases (Mb). Number of *Pf*WDR genes per chromosome is also shown in the graph.

The physical map positions of 80 *Pf*WDR genes on 14 chromosomes of *P*. *falciparum* were identified using genome browser at PlasmoDB and accordingly chromosomal localization map was constructed (Fig 8). The *Pf*WDRs were found to be widely distributed over 14 chromosomes. No gene was found to be localized at the end of chromosomes i.e. telomeric positions, where only highly variable gene families (var, rif and stevor) are known to be localized [18]. Most of the *Pf*WDR genes were located over chromosome 5 to 14. Few chromosomes had a relatively high density of *Pf*WDR genes i.e. a maximum of 12 genes were present on chromosome 12 and 13, followed by 11 genes on the longest chromosome 14.

Genes having coordinated expression and/or similar function have been shown to cluster in the *Plasmodium* genome [27]. Genomic clustering of common function genes can facilitate regulation of several genes simultaneously [44,45]. The chromosome localization map was further analyzed to determine whether genomic organization of *Pf*WDR genes were in accordance with their functions. However, no real clusters of similar function *Pf*WDRs were observed except one cluster of two genes (PF3D7_1004100, PF3D7_1004200) on chromosome number 10 (Fig 8). Further, four clusters of *Pf*WDRs leaving one/two positions in between (marked by red asterisks, Fig 8) were also present. Out of these, two were coexpressed with their proteins detected in similar stages. All *Pf*WDRs were found to be syntenic with other *Plasmodium* species except two (PF3D7_1004100, PF3D7_1428400) whose orthologs could not be detected in other *Plasmodium* species except *P*. *reichenowi* indicating towards intrasyntenic indels.

### Phylogenetic analysis

**Fig 9:**
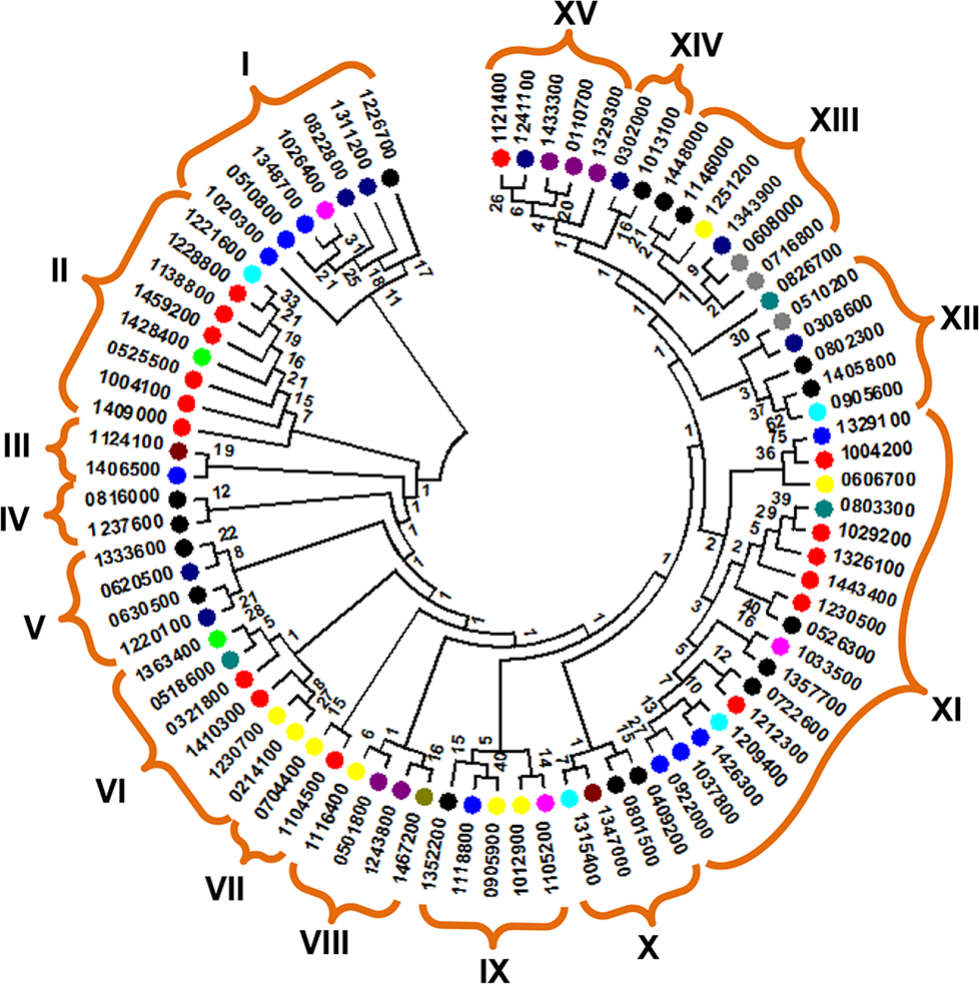
Phylogenetic relationships of the *Pf*WDRs. An un-rooted NJ tree was built using Phylip with 100 bootstrap replicates and visualized by MEGA5.2. Number at the nodes represents bootstrap values. PlasmoDB gene IDs are shown by last seven digits only along with distinctly coloured circles representing functional categories of each *Pf*WDR (see Fig 5).

To explore the evolutionary relationships of the *Pf*WDR genes, an un-rooted neighbor-joining (NJ) phylogenetic tree was generated from the alignments of full-length protein sequences as mentioned in the methods. For statistical reliability, we conducted bootstrap analysis with 100 replicates. Nevertheless, phylogenetic relationships were not much clear with very poor bootstrap values especially in internal nodes (Fig 9).

Low boot strap values were also evident in the phylogenetic analysis of foxtail millet [13]. This may be because of divergence in the WDRs resulting from variations in length, number and position of repeats, sequence conservation and domain composition; thus making it difficult to draw phylogenetic relationships. However, in outer nodes WDRs have better resolution. Accordingly, we divided the *Pf*WDRs in 15 distinct groups. Cluster XI was the largest with 17 members. Phylogenetic analysis of the WDRs of rice, *Arabidopsis* and foxtail millet divided these proteins in 5 clusters [3,12,13]. As depicted in the Fig 9, *Pf*WDRs revealed some clustering of similar function genes. For example, cluster I was mainly composed of RNA processing and cytoskeleton regulatory proteins of approximately similar length and number of WD40 repeats. Cluster II contains 6 *Plasmodium* specific WDRs with no assigned orthologs. Three chromatin assembly factors were grouped together in cluster XV. Cluster IV and V showed grouping of *Pf*WDRs involved in RNA processing. While most of the cytoskeleton regulatory *Pf*WDRs were clustered in groups I and XI.

### Homology modeling

**Fig 10:**
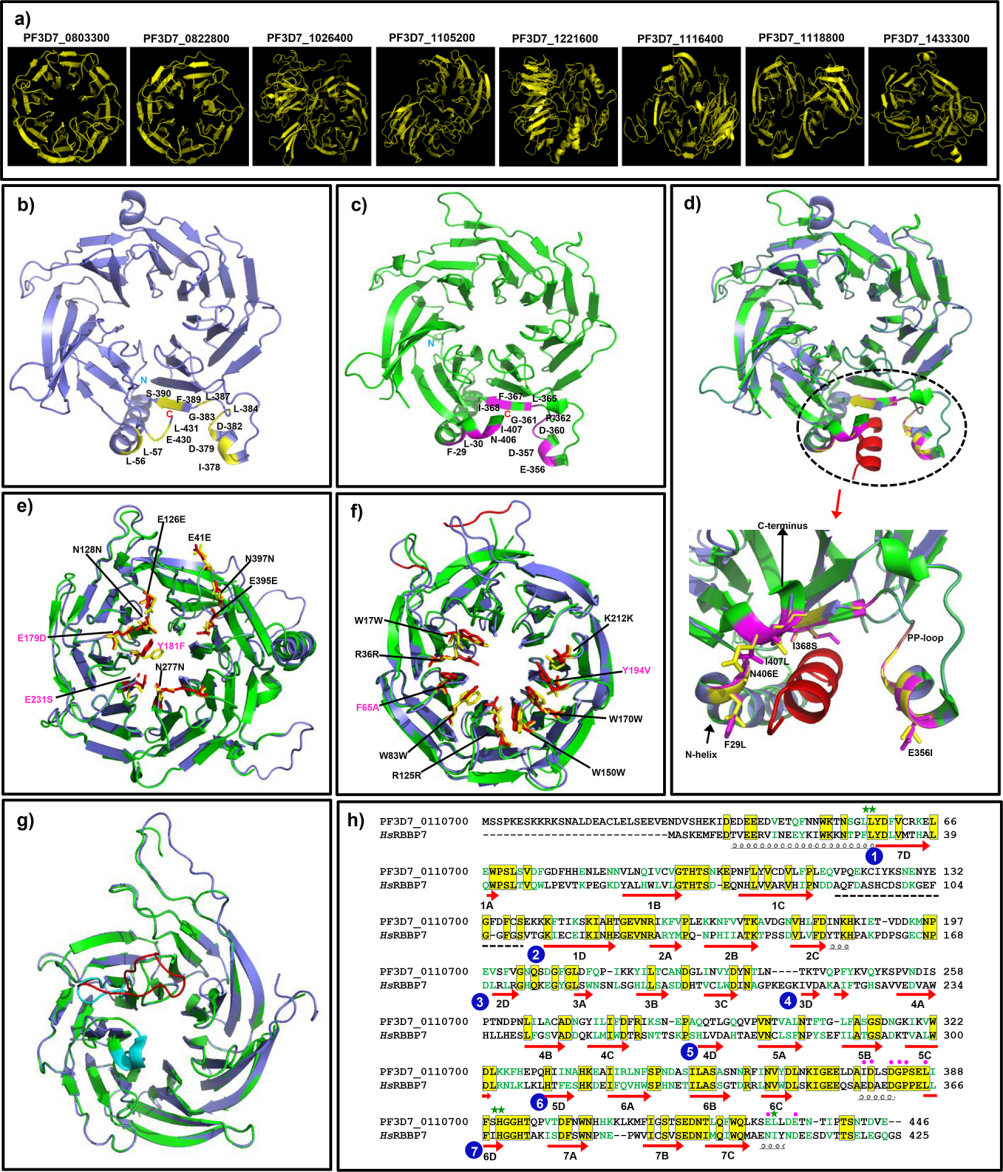
Structural analysis of the *Pf*WDRs. a) Predicted structures of the 8 *Pf*WDRs by homology modeling with >90% confidence level and ≥95% residues in the allowed region of Ramachandran plot b) Predicted 3D structure of *Pf*CAF-1 subunit (PF3D7_0110700) depicting histone H4 binding residues (yellow) as inferred from its human homolog RBBP4/RBBP7. c) Structure of *Hs*RBBP7 [5] [PDB: 3CFV, green] highlighting H4 binding residues (magenta). d) Superimposition of structures of *Pf*CAF-1 subunit and *Hs*RBBP7 with histone H4 peptide (red) clearly showing overlapping histone H4 binding pockets (highlighted in dotted circle). Close-up view of overlapped histone binding pockets is also shown depicting variant residues (highlighted as sticks) of *Pf*CAF-1 in comparison to *Hs*RBBP7. Residues position in the figure are according to *Hs*RBBP7 e.g. F29L represents Phe at 29^th^ position of *Hs*RBBP7 is replaced by Leu in *Pf*CAF-1. e) Overlay of *Pf*CAF-1 model (light blue) and *Hs*RBBP4 [6] crystal structure [PDB: 2XU7, green] highlighting FOG-1 binding residues as yellow and red sticks respectively. Residues position scheme as mentioned above. f) Structural alignment of 3D model of *Pf*RACK (PF3D7_0826700-light blue) and *Hs*RACK1 [7] [PDB: 4AOW, green]. The residues of hydrophobic ring important in binding to protein ligands at the top surface of propeller structure are shown as yellow and red sticks for *Pf*RACK and *Hs*RACK1 [7], respectively. Insertions in *Pf*RACK are highlighted in red that mainly lie in the loop regions. g) Overlay of predicted model of *Pf*WDR92 (PF3D7_1347000-light blue) with the crystal structure of *Hs*WDR92/Monad [PDB: 3I2N, green] comparing loops with insertion in *P*. *falciparum* i.e. *Pf* long loop (red) and *Hs* short loop (cyan). h) A structure based sequence alignment between *Pf*CAF-1 and *Hs*RBBP7. Secondary structure elements of *Hs*RBBP7 are shown below the alignment indicated by coils, arrows and gaps for helices, β-strands and loops, respectively [5]. Green star and magenta boxes above the alignment indicate key residues involved in hydrophobic and hydrophilic interactions with histone H4, respectively [5]. Conserved residues are highlighted in yellow boxes while similar residues are highlighted in green text. Black dotted line below alignment indicates sequence part for which no structure is available.

We employed the homology modeling approach to predict 3D structures for all *Pf*WDRs by Phyre2 or Swiss-model. Structures for only 23 *Pf*WDRs could be modeled at >90% confidence covering 80–100% residues (Fig 10, S3 Fig, S6 Table). Stereo-chemical qualities of the generated protein models were evaluated using RAMPAGE showing ~84–98% residues in allowed regions of the Ramachandran plot. As expected, 3D structures of most of the *Pf*WDRs primarily have β-propeller structures composed of β-sheets (Fig 10). Importantly, modeled structures revealed more WD40 repeats as compared to the number of WD40 repeats predicted either by SMART, Pfam or HMM. This trend was also reported by the solved crystal structures of WDR proteins [1,8] revealing more WD40 repeats as compared to WD40 repeats identified by sequence-based algorithms which may be due to the poor level of sequence conservation. Further, we could observe insertions in the *Pf*WDRs as compared to their human orthologs. However these insertions, as observed in *Pf*CAF-1 (PF3D7_0110700), *Pf*RACK (PF3D7_0826700) and *Pf*WDR92 (PF3D7_1347000) were either at N-terminus, C-terminus and loop regions that do not disturb the overall secondary structures (Fig 10D–10H).

The modeled structure of *Pf*CAF-1 was compared to its human homolog RBBP7 (RbAp46)/ RBBP4 (RbAp48) (Fig 10B–10E). The structure of *Pf*CAF-1 (Fig 10B) showed seven bladed β-propeller conformation in agreement with *Hs*RBBP7 (Fig 10C); however, β strands 1A and 7A in blades 1 and 7 were found missing. Residues responsible for binding to the histone H4 in *Pf*CAF1 and *Hs*RBBP7 are shown in the Fig 10B and 10C. As illustrated in the Fig 10D, model of *Pf*CAF-1 overlaps with the structure of *Hs*RBBP7 with conservation in the histone binding pocket present between N-terminal α-helix and PP loop (negatively charged loop which terminates in two proline) [5]. The subtle differences (F29L, E356I, I368S, I407L and N406E) in the conserved histone binding pocket are highlighted in Fig 10D. Further, FOG-1 transcription factor binding site in *Hs*RBBP4 [6] was also found to be conserved in *Pf*CAF-1 except some differences (E179D, Y181F and E231S) (Fig 10E).

Likewise, we also compared modeled structure of *Pf*RACK with the crystal structure of *Hs*RACK1 [7]. The presence of hydrophobic ring (important in binding to protein ligands) on the top surface of *Pf*RACK showed good agreement with *Hs*RACK1 but few differences were also evident (Fig 10F). The superimposed structures of *Pf*WDR92 and *Hs*WDR92 revealed insertions in the loop region between blades (Fig 10G). Thus, the comparison of *Pf*WDRs and *Hs*WDRs revealed subtle parasite-specific structural features apparent as partial conservation of important binding residues and insertions in loop regions in *Pf*WDRs.

### Protein-protein interactions

**Fig 11:**
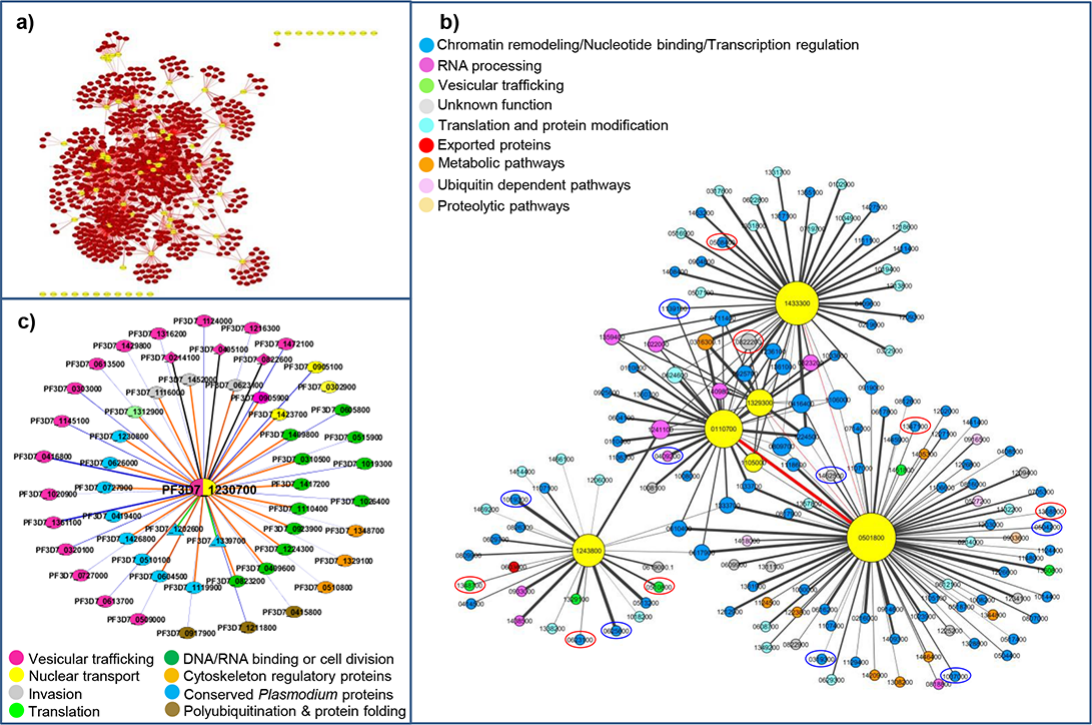
Protein-protein interactions (PPIs) network analysis for the *Pf*WDRs. a) PPIs network of all the 80 *Pf*WDRs (yellow nodes). b) PPIs network of the *Pf*WDRs predicted to be engaged in chromatin assembly and remodeling (yellow nodes). Node size is proportional to the degree of node. Nodes are coloured according to their functional classification based on PlasmoDB/human homologs annotations. Edge width is proportional to the confidence score from STRING for each interaction. Interactions among *Pf*WDR proteins are highlighted with red edges. Nodes not coexpressed even at a single stage with the *Pf*WDRs are encircled in red. The nodes for which no protein expression data was available at PlasmoDB are encircled in blue colour. c) PPIs network of *Pf*Sec13 (PF3D7_1230700) (yellow and magenta node) derived from STRING (outer ring with blue edges), co-IP [16] (inner ring with orange edges) and Y2H (triangles with green edges). Interactions common between STRING and co-IP are indicated by diamond shapes and black edges. Nodes are colour coded as per their functions.

The unifying role of most WDRs is simultaneous or sequential binding to other proteins. We mined the PPIs for the 80 *Pf*WDR proteins on the basis of experiment, text mining and database evidences available at the STRING database, Y2H datasets available at PlasmoDB [46] and individual experimental interaction evidence. Sixty out of 80 *Pf*WDR proteins were associated with at least one other protein comprising a total of 1928 PPIs depicted in Fig 11A (S7 Table). Out of these, 1847 PPIs were derived from STRING, 60 by Y2H and 25 from co-immunoprecipitation (co-IP) of HA tagged *Pf*Sec13 [16] with four overlaps i.e. one common between STRING and Y2H and three common between STRING and co-IP. The extent of connectivity differs among the 60 *Pf*WDRs with PPIs ranging from 1 (PF3D7_1347000, PF3D7_1221600) to 110 (PF3D7_0308600) (S7 Table). Five *Pf*WDRs have less than 5 interacting partners, 12 have 5–10 partners and 43 proteins were highly connected with more than 11 partners, suggesting that these proteins were involved in complex cellular networks. The confidence score of interactions derived from STRING database ranged from 0.4 (medium) to 0.9 (high) with 653 associations (35.4%) having high confidence scores (S >0.7) and 1194 associations (64.6%) having medium confidence scores (0.4≤S<0.7). As per the Y2H data, only 18 *Pf*WDRs were involved in interactions resulting in 60 PPIs ranging from 1 to 17. Nevertheless, interactions shown by Y2H do not overlap with the interactions predicted by STRING except one.

Statistical GO enrichment of the *Pf*WDRs associations using BiNGO, revealed over-representation of 138 GO ontology terms (p <0.05) (S4 Fig, S8 Table) emphasizing the involvement of *Pf*WDRs in many basic cellular, molecular and biological processes. Fig 11B shows the PPI network of 5 *Pf*WDRs involved in chromatin remodeling/ chromatin associated processes as derived from STRING with a confidence score >0.3. The interaction network was composed of 203 interactions. Amongst these, PF3D7_0501800 has maximum of 83 interactions. As anticipated, the highlight of this sub-network is involvement of 129 interactions with chromatin/transcription factors/DNA binding proteins. All the 5 *Pf*WDRs were found to interact with one or more histones which further authenticate their role in chromatin associated processes.

PPIs for *Pf*Sec13, a protein identified as component of nuclear pore complex and COPII coat, are depicted in Fig 11C. Interaction network was composed of 54 PPIs derived from STRING (30 PPIs), Y2H (2PPIs) and HA tag co-IP (25PPIs) as performed by Dahan-Pasternak et al. [16]. In this network only 3 PPIs overlapped between STRING and co-IP datasets; questioning the reliability of predictions of the STRING database in case of *Plasmodium*. Y2H dataset detected only 2 PPIs that too non-overlapping with other two datasets. This points towards Y2H assays drawbacks in terms of sensitivity and specificity. STRING predicted 7 high scoring interactions (S>0.7) for *Pf*Sec13 out of these 3 overlaps with co-IP. These 3 overlapping interacting proteins belong to coat complex of COPII vesicles i.e. sec23, sec24-like and sec31. A functional comparison of STRING PPIs and co-IP PPIs of *Pf*Sec13 is shown in Fig 11C.

## Conclusions

In the present study, an extensive analysis of the *Pf*WDRs in terms of their domain attributes, functional classification, genomic and subcellular localization, transcript profiles, protein expression, evolutionary relations, sequence features, homology modeling and interaction networks was performed. This study led to the identification of 80 putative *Pf*WDRs. Analysis of the Pfam and *P*. *falciparum* WDR logos highlighted poor sequence conservation of the WD40 motif. A larger fraction of *P*. *falciparum* proteome is devoted to WDRs as compared to humans. Of note, we have identified 5 distinct *Pf*WDRs with no clear human counterparts in terms of their domain structures. Importantly, assignment of orthologs to the *Pf*WDRs helped us to annotate and predict potential functions of several *Pf*WDRs. Interestingly, most of the assigned human orthologs were of shorter length as compared to their respective *Pf*WDRs underscoring the presence of residue insertions in *Plasmodium* proteins. The phylogenetic analysis hints at divergence in *Pf*WDRs as no clear evolutionary relationships could be drawn within the *Pf*WDR superfamily. Proteome profiling revealed presence of most of the *Pf*WDRs in multiple stages of *Plasmodium* life cycle except for 9 *Pf*WDRs that are restricted to single stage either of S, M, G, Sp. Expression profiling disclosed a blend of linear and nonlinear correlations between mRNA and protein existence as well as abundance between various *Plasmodium* life cycle stages. Our efforts to draw relationships between transcriptome and proteome profiles of the *Pf*WDRs are an important addition to a handful of similar studies [40–43]. Our analyses of the modeled *Pf*WDR 3D structures highlighted slight deviations in highly conserved binding sites and presence of insertions mainly in loop regions. PPI network analysis suggested the involvement of *Pf*WDRs in a large number of interactions. In summary, the present efforts to identify and describe key attributes of this uncharacterized WDR family in *P*. *falciparum* provide a foundation for dissection of their regulatory roles in parasite biology.

## Methods

### Extraction of putative *Pf*WDR genes and domain composition analysis

Two approaches were used for the mining of *Plasmodium* genomic resource database PlasmoDBv9.0 (http://plasmodb.org/plasmo/) [47] to obtain all the putative *Pf*WDR genes. Firstly, PlasmoDB was explored by text search using keyword ‘WD40’ and InterPro domain search ‘IPR001680: WD40_repeat, IPR017986: WD40_repeat_dom’ [48]. Secondly, HMM search was performed with the WD40 domain HMM seed (PF00400) downloaded from Pfam [49] against *P*. *falciparum* protein database using HMMER3.0 program [50]. To confirm the presence of WD40 domain in each of the predicted *Pf*WDR, Pfam and SMART databases [19] were explored. Domain architecture for the *Pf*WDRs was drawn manually as identified by Pfam, SMART and InterPro. Sequence logos were generated using Skylign tool [51].

### Ortholog and functional assignments

Each *Pf*WDR protein sequence was queried against UniProtKB or non redundant protein sequence databases using NCBI BLAST/PSI-BLAST as well as against organism specific databases. The hits were explored manually for the assignment of putative orthologs based on a number of parameters i.e. e-value and score, sequence coverage, percentage identity, the length of the hit as compared to the query, domain composition, UniProt annotation for the protein, any functional knowledge and available literature review. Additionally, OrthoMCL [52] and PhylomeDB [53] were also queried for the identification of putative orthologs.

Functional classification of the *Pf*WDRs was done manually based on their annotation in PlasmoDB and conservation of this annotation in various *Plasmodium* species and/or annotation of their orthologs at UniPort or their respective databases, related literature either for *Pf*WDR or its homolog or for a particular functional annotation/category with a further assistance from Gene Ontology database (www.geneontology.org).

### Subcellular localization and expression profiling of *Pf*WDRs

To predict subcellular location of the *Pf*WDRs, firstly, experimental evidences either for *Pf*WDR or its ortholog through ApiLoc v3 (http://apiloc.biochem.unimelb.edu.au) and published articles were searched. Additionally, various online servers like MitoProt [54], Euk-mPLoc 2.0 server [55], PATS [56], PlasmoAP [57], PSORT II [58] and NetNES [59] were also explored. Evaluation of the transcriptomic data [25,26] and proteomic data [27–37] of *Pf*WDRs obtained from literature was done using MeV (version 4.9) software. For Le Roch et al. [26] transcriptome data, K-means clustering was performed with MeV using 4 classes and Euclidian distance of genes.

### Phylogenetic analysis and homology modeling of *Pf*WDRs

Multiple sequence alignment of the full length *Pf*WDRs was carried out using Clustal Omega (http://www.ebi.ac.uk/Tools/msa/clustalo/). Subsequently, an un-rooted NJ tree was constructed using the program Phylip v3.695 [60] with standard parameters and visualized by MEGA v5.2 [61].

3D structures of *Pf*WDRs were predicted by homology modeling servers Phyre2 [62] and Swiss-model [63]. Structures were visualized with PYMOL [64]. Structural validation of the protein models was done using RAMPAGE [65] and QMEAN server available at Swiss- model [63].

### Network data analysis

The entire set of protein-protein interactions for 80 *Pf*WDRs was extracted from the STRING database [66] having a confidence score (S) in a range of 0.4 to 0.999 based on experiment, text mining and database evidences. In addition, Y2H datasets [46] and pull downs and any other experimental interaction data of *Pf*WDRs through literature were also explored. Undirected weighted graph with a single edge between any pair of proteins weighed by the S value was generated in Cytoscape [67]. BinGO application within Cytoscape was employed to identify enriched GO terms using hypergeometric test with Benjamini and Hochberg FDR correction at 0.05 significance level. Y2H interaction data was obtained from PlasmoDB.

## Supporting Information

S1 Fig Clustering of Le Roch et al. [26] data. The data was clustered in four groups from low to high expression (blue-yellow colorimetric representation) using Mev 4.9. Different stages of IDC are as in Fig 7. Gene IDs on right side are coloured according to their functional classification (see Fig 5).
(TIFF)

S2 Fig Expression patterns of stage specific *Pf*WDR genes confined to one or two stages. Figure legend is same as of Fig 7. Genes having coordination between transcriptome and proteome are marked with asterisk.
(TIF)

S3 Fig Predicted structures of the 12 *Pf*WDRs by homology modeling with >90% confidence level and 85–94% residues in the allowed region of Ramachandran plot. (TIF)

S4 Fig Graphical representation of Gene Ontology analysis based on molecular, cellular and biological processes using BiNGO. The size of node is proportional to the number of proteins represented by GO term. The colour represents the enrichment significance (p-value) for each GO term while white nodes are not enriched and represents the hierarchical relationship among enriched members.
(TIFF)

S1 File *Pf*WDRs sequences alignment (.sto file) (File A) and seed HMM file (.hmm file) (File B). (ZIP)

S1 Table List of putative *Pf*WDR genes. List of 80 putative *Pf*WDR genes confirmed by SMART and/or Pfam databases (**Table A**). List of 12 *Pf*WDR genes in which WD40 domain is confirmed by Superfamily database only (**Table B**). Information regarding product description, gene location, nucleotide sequence length, number of introns, isoelectric point, molecular weight and amino acid sequence length as extracted from PlasmoDB.
(XLSX)

S2 Table Data file for the graph in Fig 1C showing the predicted percentage of proteome of eukaryotic organisms devoted for the WDR proteins. (DOCX)

S3 Table Functional classification and human orthologs of the *Pf*WDRs. Domains specific to *P*. *falciparum* and *H*. *sapiens* are indicated with superscript ‘Pf’ and ‘Hs’. Orthologs in other organisms are given in ‘[]’ where human homologs were traced based on these.
(DOC)

S4 Table Orthologs of the *Pf*WDRs. List of *B*. *bovis* and *T*. *gondii* orthologs of 61 *Pf*WDRs (with assigned human homologs) (**Table A**). Orthologs of 19 *Pf*WDRs (with no assigned human homolog) in various organisms i.e. *Plasmodium vivax*, *P*. *berghei*, *Tetrahymena thermophila*, *B*. *bovis*, *T*. *gondii*, *Cryptosporidium parvum*, *Theileria annulata*, *S*. *cerevisiae*, *Drosophila melanogaster*, *A*. *thaliana*, *H*. *sapiens* (**Table B**). Doubtful orthologs are given in grey text.
(XLSX)

S5 Table Predicted subcellular localization of the *Pf*WDRs derived from various online programs and published sources (either for *Pf*WDRs or assigned orthologs). Localization of gene IDs marked with asterisk was predicted *in silico*.
(XLSX)

S6 Table Characteristics of the homology modeled 3D structures of 23 *Pf*WDRs. (DOCX)

S7 Table *Pf*WDRs protein-protein associations derived from STRING database, Y2H interaction data and literature. (XLSX)

S8 Table BinGO results for over-represented GO functional categories. (XLSX)
