# Supplementary figures and images for: Genome-Wide Collation of the Plasmodium falciparum WDR Protein Superfamily Reveals Malarial Parasite-Specific Features
Source: PLoS One. 2015 Jun 4;10(6):e0128507. doi: 10.1371/journal.pone.0128507 (PMC4456382; doi:10.1371/journal.pone.0128507)

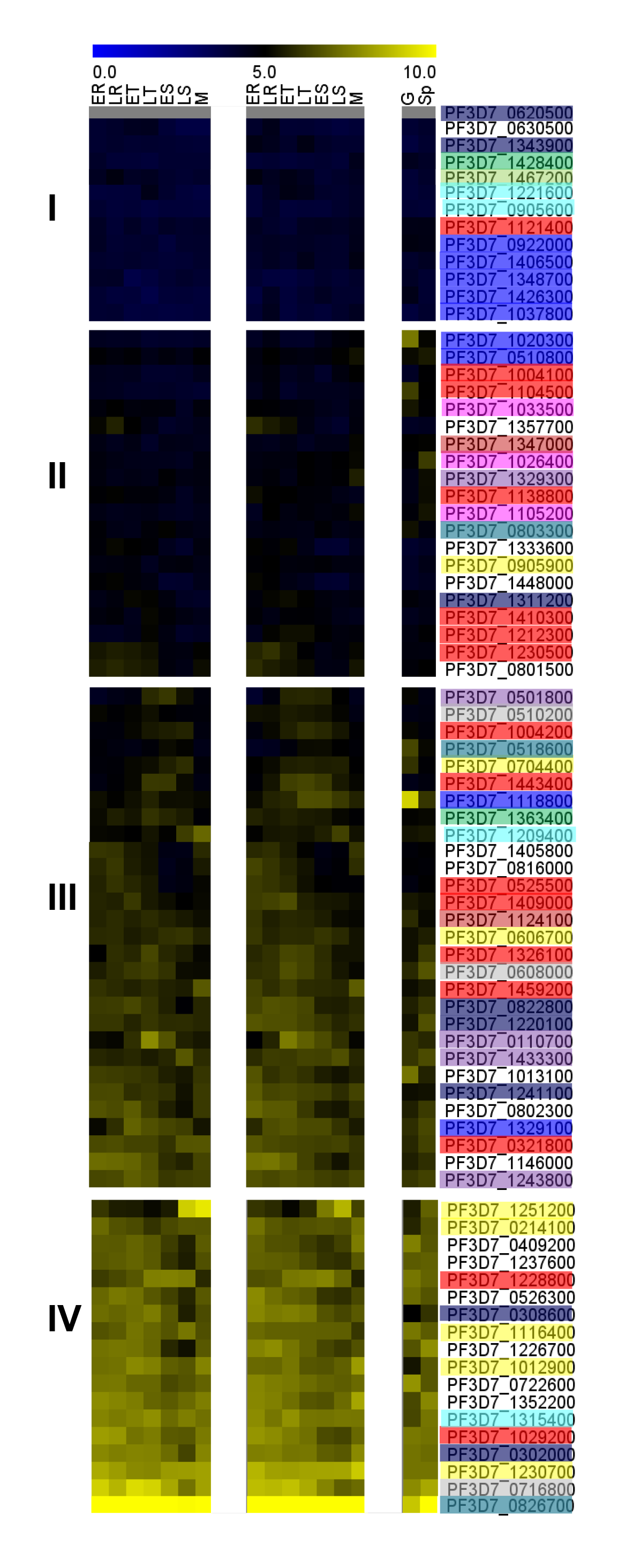

Supplement: S1 Fig — The data was clustered in four groups from low to high expression (blue-yellow colorimetric representation) using Mev 4.9. Different stages of IDC are as in Fig 7. Gene IDs on right side are coloured according to their functional classification (see Fig 5). (TIFF) [file pone.0128507.s001.tiff]

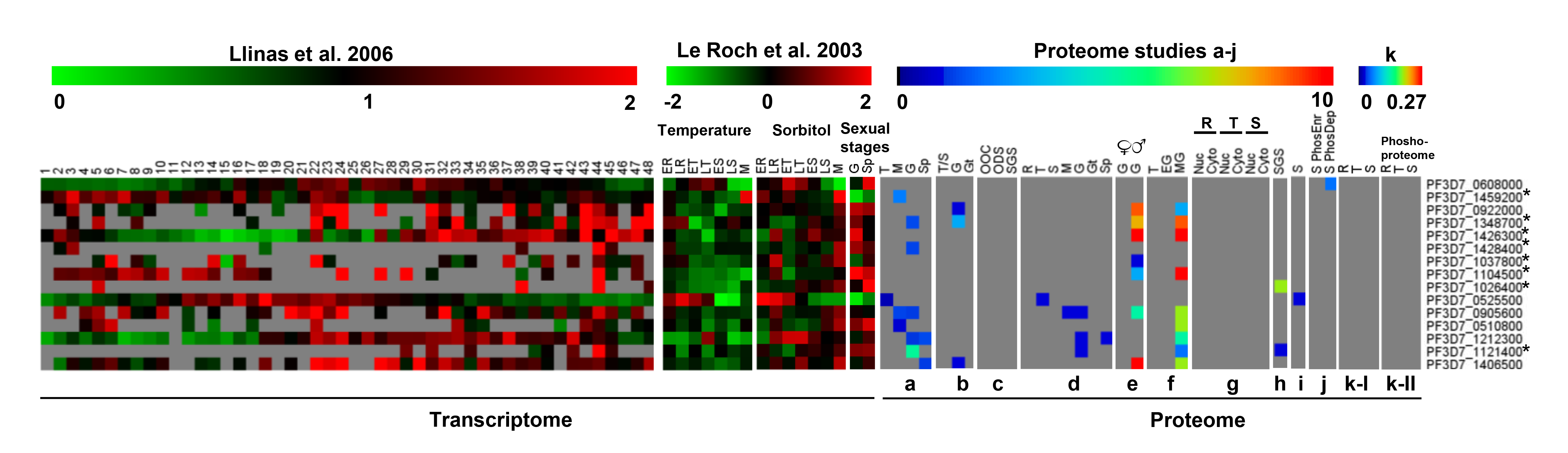

Supplement: S2 Fig — Figure legend is same as of Fig 7. Genes having coordination between transcriptome and proteome are marked with asterisk. (TIF) [file pone.0128507.s002.tif]

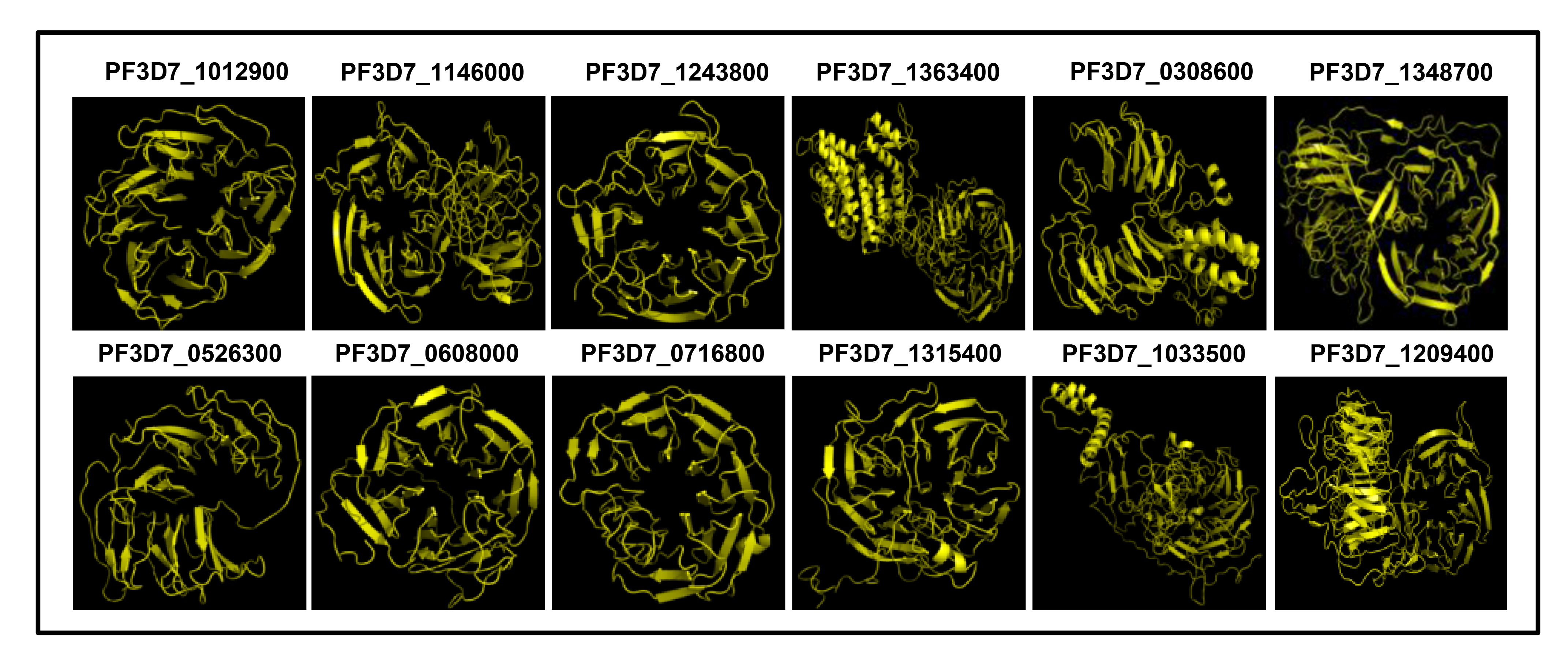

Supplement: S3 Fig — (TIF) [file pone.0128507.s003.tif]

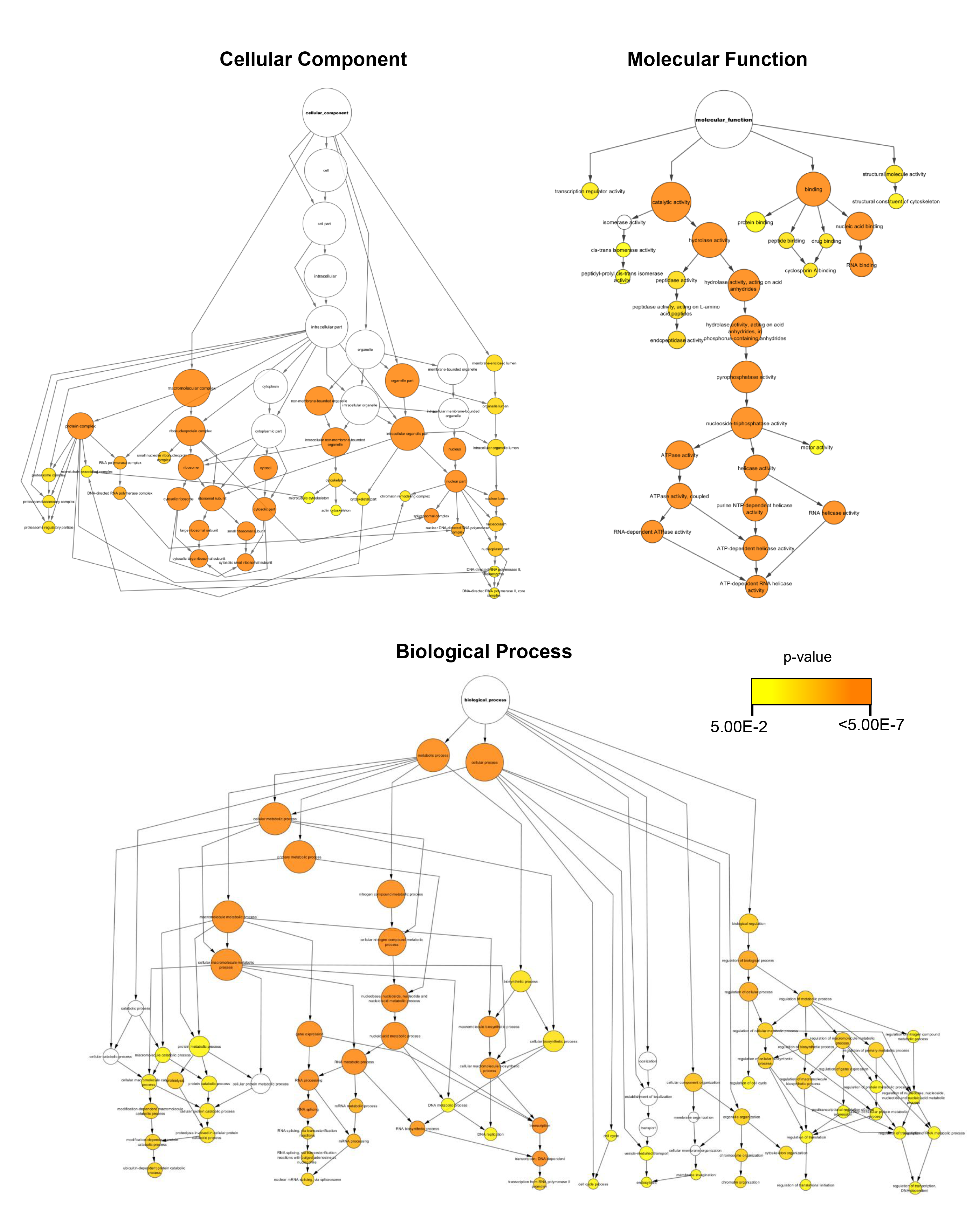

Supplement: S4 Fig — The size of node is proportional to the number of proteins represented by GO term. The colour represents the enrichment significance (p-value) for each GO term while white nodes are not enriched and represents the hierarchical relationship among enriched members. (TIFF) [file pone.0128507.s004.tiff]
