# Supplementary material for: Genome-Wide Collation of the Plasmodium falciparum WDR Protein Superfamily Reveals Malarial Parasite-Specific Features
Source: PLoS One. 2015 Jun 4;10(6):e0128507. doi: 10.1371/journal.pone.0128507 (PMC4456382; doi:10.1371/journal.pone.0128507)
Supplement: S2 Table — (DOCX) [file pone.0128507.s007.docx]

**Table S2. Data file for the graph in Fig. 1c showing the predicted percentage of proteome of eukaryotic organisms devoted for the WDR proteins.**

| **Sr. No.** | **Organism** | **Number of WDR proteins** | **Source** | **Proteome size** | **Source** | **Percentage of WDR proteins in proteome** |
| --- | --- | --- | --- | --- | --- | --- |
| 1 | *Cryptosporidium hominis* | 80 | Text search /IPR017986 | 3886 | [CryptoDB.org](http://cryptodb.org/cryptodb/) | 2.06 |
| 2 | *Cryptosporidium parvum* | 88 | Text search /IPR017986 | 3805 | [CryptoDB.org](http://cryptodb.org/cryptodb/) | 2.31 |
| 3 | *Eimeria tenella* | 99 | Text search /IPR017986 | 8597 | [ToxoDB.org](http://toxodb.org/toxo/) | 1.15 |
| 4 | *Toxoplasma gondii* | 126 | Text search /IPR017986 | 8460 | [ToxoDB.org](http://toxodb.org/toxo/) | 1.49 |
| 5 | *Plasmodium falciparum* | 92 | Text search /IPR017986 | 5398 | [PlasmoDB.org](http://plasmodb.org/plasmo/) | 1.70 |
| 6 | *Plasmodium vivax* | 96 | Text search /IPR017986 | 5530 | [PlasmoDB.org](http://plasmodb.org/plasmo/) | 1.73 |
| 7 | *Plasmodium knowlesi* | 97 | Text search /IPR017986 | 5224 | [PlasmoDB.org](http://plasmodb.org/plasmo/) | 1.85 |
| 8 | *Plasmodium berghei* | 91 | Text search /IPR017986 | 4908 | [PlasmoDB.org](http://plasmodb.org/plasmo/) | 1.85 |
| 9 | *Theileria parva* | 75 | Text search /IPR017986 | 4082 | [PiroplasmaDB.org](http://piroplasmadb.org/piro/) | 1.84 |
| 10 | *Theileria annulata* | 76 | Text search /IPR017986 | 3795 | [PiroplasmaDB.org](http://piroplasmadb.org/piro/) | 2 |
| 11 | *Babesia bovis* | 82 | Text search /IPR017986 | 3706 | [PiroplasmaDB.org](http://piroplasmadb.org/piro/) | 2.21 |
| 12 | *Tetrahymena thermophila* | 344 | SMART | 26996 | [Ciliate .org](http://ciliate.org/index.php/home/welcome) | 1.27 |
| 13 | *Trypanosoma brucei* | 140 | SMART | 8309 | [TritrypDB.org](http://tritrypdb.org/tritrypdb/) | 1.68 |
| 14 | *Leshmania infantum* | 124 | SMART | 8180 | [TritrypDB.org](http://tritrypdb.org/tritrypdb/) | 1.51 |
| 15 | *Dictyostelium discoideum* | 181 | SMART | 12258 | [dictybase.org](http://dictybase.org/) | 1.47 |
| 16 | *Neurospora crassa* | 138 | Text search /IPR017986 | 10812 | [Fungidb.org](http://fungidb.org/fungidb/) | 1.276 |
| 17 | *Aspergillus niger* | 144 | Text search /IPR017986 | 14070 | [Fungidb.org](http://fungidb.org/fungidb/) | 1.02 |
| 18 | *Saccharomyces cerevisiae* | 116 | Text search /IPR017986 | 6602 | [Fungidb.org](http://fungidb.org/fungidb/) | 1.76 |
| 19 | *Caenorhabditis elegans* | 140 | SMART | 20513 | [wormbase.org](http://www.wormbase.org/#01-23-6) | 0.68 |
| 20 | *Anopheles gambiae* | 177 | SMART | 12843 | [vectorbase.org](https://www.vectorbase.org/) | 1.38 |
| 21 | *Drosophila melanogaster* | 187 | SMART | 13972 | [flybase.org](http://flybase.org/) | 1.34 |
| 22 | *Chlamydomonas reinhardtii* | 179 | SMART | 14337 | [Uniprot.org](http://www.uniprot.org/) | 1.25 |
| 23 | *Zea mays* | 365 | SMART | 57643 | [Uniprot.org](http://www.uniprot.org/) | 0.63 |
| 24 | *Sorghum bicolor* | 223 | SMART | 32796 | [Uniprot.org](http://www.uniprot.org/) | 0.68 |
| 25 | *Setaria italica* | 225 | [1] | 39110 | [Uniprot.org](http://www.uniprot.org/) | 0.57 |
| 26 | *Vitis vinifera* | 275 | SMART | 29971 | [vtcdb.adelaide.edu.au](http://vtcdb.adelaide.edu.au/Home.aspx) | 0.92 |
| 27 | *Oryza sativa (japonica)* | 200 | [2] | 39102 | [rice.plantbiology.msu.edu](http://rice.plantbiology.msu.edu/) | 0.51 |
| 28 | *Arabidopsis thaliana* | 237 | [3] | 27416 | [arabidopsis.org](http://www.arabidopsis.org/) | 0.86 |
| 29 | *Danio rerio* | 272 | SMART | 26459 | [ensemble.org](http://www.ensembl.org/Danio_rerio/Info/Annotation/) | 1.03 |
| 30 | *Ciona intestinalis* | 196 | SMART | 17308 | [Uniprot.org](http://www.uniprot.org/) | 1.13 |
| 31 | *Gallus gallus* | 220 | SMART | 17623 | [Uniprot.org](http://www.uniprot.org/) | 1.25 |
| 32 | *Canis lupus familiaris* | 264 | SMART | 25441 | [Uniprot.org](http://www.uniprot.org/) | 1.04 |
| 33 | *Rattus norvegicus* | 258 | SMART | 23363 | [rgd.mcw.edu](http://rgd.mcw.edu/) | 1.10 |
| 34 | *Mus musculus* | 262 | SMART | 24551 | <http://informatics.jax.org/> | 1.07 |
| 35 | *Homo sapiens* | 267 | SMART | 21785 | [4] | 1.23 |

**References**

1. Mishra AK, Muthamilarasan M, Khan Y, Parida SK, Prasad M (2014) Genome-wide investigation and expression analyses of WD40 protein family in the model plant foxtail millet (*Setaria italica L.).* PLoS One 9: e86852.
2. Ouyang Y, Huang X, Lu Z, Yao J (2012) Genomic survey, expression profile and co-expression network analysis of OsWD40 family in rice. BMC Genomics 13: 100.
3. van Nocker S, Ludwig P (2003) The WD-repeat protein superfamily in Arabidopsis: conservation and divergence in structure and function. BMC Genomics 4: 50.
4. Schad E, Tompa P, Hegyi H (2011) The relationship between proteome size, structural disorder and organism complexity. Genome Biol 12: R120.
