# Supplementary material for: Genome-Wide Collation of the Plasmodium falciparum WDR Protein Superfamily Reveals Malarial Parasite-Specific Features
Source: PLoS One. 2015 Jun 4;10(6):e0128507. doi: 10.1371/journal.pone.0128507 (PMC4456382; doi:10.1371/journal.pone.0128507)
Supplement: S3 Table — Domains specific to P. falciparum and H. sapiens are indicated with superscript ‘Pf’ and ‘Hs’. Orthologs in other organisms are given in ‘[]’ where human homologs were traced based on these. (DOC) [file pone.0128507.s008.doc]

**Table S3. Functional classification of the *Pf*WDRs. Domains specific to *P. falciparum* and *H. sapiens* are indicated with superscript ‘Pf’ and ‘Hs’. Orthologs in other organisms are given in ‘[ ]’ where human orthologs were traced based on these.**

| **Function** | | | | **Gene ID’s of *Pf*WDRs** | **Description of *Pf*WDRs** | **Human orthologs** | | | | **Domain composition** | **Protein length (aa -*P. falciparum* / Human)** | **References** |
| --- | --- | --- | --- | --- | --- | --- | --- | --- | --- | --- | --- | --- |
|  |  |  |  |  |  | **Gene ID** | **Description** | **E-value** | **% Coverage / % Identity** |  |  |  |
| **Chromatin assembly and chromatin remodeling proteins** | | | | PF3D7_0110700 | Chromatin assembly factor 1 protein WD40 domain, putative | NP_005601.1/NP_002884.1 | RBBP4/RBBP7 | 2e-57 or 2e-55 | 92/31 or 91/31 | CAF1C H4-bd + WD40 | 446/425 | [1-3] |
|  |  |  |  | PF3D7_1329300 | Chromatin assembly factor 1 subunit, putative | NP_005601.1 | RBBP4 | 1e-32 | 93/30 | CAF1C_H4-bd + WD40 | 582/425 | [3] |
|  |  |  |  | PF3D7_1433300 | Chromatin assembly factor 1 P55 subunit, putative | NP_005601.1/NP_002884.1 | RBBP7/RBBP4 | 3e-72 or 3e-71 | 93/31 or 90/31 | CAF1C H4-bd + WD40 | 428/425 | [2,4] |
|  |  |  |  | PF3D7_0501800 | Chromosome assembly factor 1 (CAF1) | **NP_005432.1**  **/NP_003316.3** | CAF-1 subunit B or Protein HIRA | 2e-12 or 1e-17 | 23/28 or 16/33 | WD40 + CAF-1-p60_C^Hs^ /Hira^Hs^ | 1076/559 or 1076/ 1017 | [3] |
|  |  |  |  | PF3D7_1243800 | Microtubule associated katanin, putative | NP_079498.2 | WDR 82 | 3e-35 | 87/29 | WD40 | 370/313 | [5-7] |
| **Vesicular trafficking** | | | **a) Protein transport proteins (CopII proteins )** | PF3D7_1230700 | Protein transport protein Sec13, putative | NP_899195.1 | Protein SEC13 homolog | 9e-46 | 42/35 | WD40 | 822/322 | [3,8,9] |
|  |  |  |  | PF3D7_0214100 | Protein transport protein sec31 | NP_001070674.1 | Protein transport protein Sec31A | 8e-37 | 67/31 | Sec16_C + WD40 | 1471/1220 | [3,10-12] |
|  |  |  |  | PF3D7_1116400 | ER membrane protein Sec12 | NP_037520.1 | Prolactin regulatory element-binding protein (Sec12) | 7e-06 | 59/20 | WD40 +  Transmembrane | 485/417 | [13] |
|  |  |  | **b**) **Coatomer complex (CopI proteins)** | PF3D7_0606700 | Coatomer alpha subunit, putative | NP_004362.2 | Coatomer subunit alpha | 6e-76 | 73/45 | Coatomer_WDAD + COPI_C + WD40 | 1512/1224 | [3,14] |
|  |  |  |  | PF3D7_0905900 | Beta subunit of coatomer complex, putative | NP_004757.1 | Coatomer subunit beta | 0 | 87/38 | Coatomer_WDAD + WD40 | 1010/906 | [3,15] |
|  |  |  | **c) Coronin** | PF3D7_1251200 | Coronin | NP_006082.3 | Coronin-2B | 6e-57 | 68/32 | WD40 + DUF1899 + DUF1900 | 602/480 | [3,16] |
|  |  |  | **d) Others** | PF3D7_0704400 | Phosphoinositide-binding protein, putative | _ | _ | _ |  | WD40+ PX | 1010/- | [3] |
|  |  |  |  | PF3D7_1012900 | Autophagy-related protein 18, putative | NP_062559.2 / NP_060453.2 | WD repeat domain phosphoinositide-interacting protein 3 (WIPI3) / WIPI1 | 2e-57 / 2e-28 | 93/32 or 96/27 | WD40 | 380/344 or 380/446 | [3,17-20] |
| **Signal transduction** | | | | PF3D7_0826700 | Receptor for activated c kinase (RACK)  (Ribosomal protein-translation) | NP_006089.1 | Receptor for activated C kinase | 6e-129 | 95/60 | WD40 | 323/317 | [3,21-23] |
|  |  |  |  | PF3D7_0803300 | Mitogen-activated protein kinase organizer 1, putative (*Pf*MORG1)  (Signaling and cell polarization) | NP_115708.1 | Mitogen-activated protein kinase organizer 1(WDR83) | 2e-29 | 66/30 | WD40 | 365/315 | [24,25] |
|  |  |  |  | PF3D7_0518600 | WD-repeat protein, putative | NP_079436.3 | WD repeat-containing protein 26 (WDR26) or CUL4- and DDB1-associated WDR protein 2 | 1e-40 | 42/33 | LisH + WD40 + CTLH^Hs^ | 1276/661 | [26,27] |
| **Cell division** | | | | PF3D7_1026400 | Cell division cycle protein 20 homolog, putative  (Male gametocyte division and differentiation) | NP_057347.2 | Fizzy-related protein homolog Or CDC20-like protein 1 | 9e-84 | 61/37 | WD40 | 603/496 | [28-32] |
|  |  |  |  | PF3D7_1105200 | Conserved Plasmodium protein, unknown function | NP_060288.2 | WD repeat containing protein WRAP73 or(wdr8) (Constituent of centrosome and localized to spindle pole bodies (SPBs)) | 7e-23 | 91/25 | WD40 | 463/460 | [33-35] |
|  |  |  |  | PF3D7_1033500 | WD-repeat protein, putative | NP_060504.1 | WD repeat-containing protein 70 (WDR70)  (*C. elegans* homolog GAD1(O16519) - involved in gastrulation and mitotic spindle orientation) | 3e-23 | 67/24 | WD40 | 709/654 | [36] |
| **Telomere replication** | | | | PF3D7_1467200 | Conserved Plasmodium protein, unknown function | NP_060551.1 | Telomerase Cajal body protein 1 Or WDR79 or WRAP53 | 9e-30 | 62/26 | WD40 | 579/548 | [37-39] |
| **Apoptosis** | | | | PF3D7_1347000 | G-beta repeat protein , putative | NP_612467.1 | Monad or WDR 92 | 6e-67 | 99/37 | WD40 | 363/357 | [40-42] |
|  |  |  |  | PF3D7_1124100 | Neutral-sphingomyelinase activation factor protein, putative  (TNF receptor signaling) | NP_003571.2 | Factor associated with neutral sphingomyelinase activation | 3e-69 | 26/38 | Beach + WD40 +  GRAM^Hs^ | 1653/917 | [43,44] |
| **Ubiquitin-dependent protein catabolism** | | | | PF3D7_1363400 | Polyubiquitin binding protein, putative | NP_001026859.1 | Phospholipase A-2-activating protein(PLA2P) | 2e-21 | 78/21 | PFU + PUL + WD40 | 905/795 | [45-47] |
|  |  |  |  | PF3D7_1428400 | Probable protein, unknown function | NP_055838.2 | WD and tetratricopeptide repeats protein 1 or (DCAF9) | 5e-23 | 28/24 | WD40 + TPR | 1990/677 | [48,49] |
| **RNA processing** | **a) rRNA processing and Ribosome biogenesis proteins** | | | PF3D7_0802300 | rRNA processing WD-repeat protein, putative | NP_005040.2 | Periodic tryptophan protein 2 homolog | 1e-57 | 81/26 | UTP12 + WD40 | 1121/919 | [50,51] |
|  |  |  |  | PF3D7_1448000 | U3 snoRNA-associated small subunit rRNA processing protein, putative | NP_006775.1 | WD repeat-containing protein 3 (WDR3) | 2e-35 | 49/32 | WD40 + UTP12^Hs^ | 1275/943 | [50,52 ] |
|  |  |  |  | PF3D7_1013100 | U3 snoRNA-associated small subunit rRNA processing protein, putative | NP_006444.2 | Transducin beta-like protein 3 (TBL3) | 4e-21 | 39/22 | UTP13 + WD40 | 1220/808 | [50,51,52] |
|  |  |  |  | PF3D7_1352200 | Conserved Plasmodium protein, unknown function | NP_115551.2  [*T. annulata -* TA16915] | U3 small nucleolar RNA-associated protein 15 homolog [Putative uncharacterized protein] | 4e-09  [4e-20] | 52/20  [88/21] | WD40 + UTP15_C^Hs^  [WD40 + UTP15_C^Ta^] | 464/518  [464/489] | [50] |
|  |  |  |  | PF3D7_1357700 | U3 snoRNA-associated small subunit rRNA processing protein, putative | NP_644810.1 | WD repeat-containing protein 36 (WDR36) | 5e-12 | 72/20 | UTP21 + WD40 | 1218/951 | [3,50,55] |
|  |  |  |  | PF3D7_0722600 | Nucleolar rRNA processing protein, putative | NP_005443.3 | WD repeat-containing protein 46 (WDR46) | 5e-62 | 61/33 | BING4CT + WD40 | 602/610 | [56-58] |
|  |  |  |  | PF3D7_0409200 | 40S ribosomal processing protein, putative | NP_056235.3 | WDSOF1 or DDB1 and CUL4 associated factor 13 (DCAF13) | 3e-120 | 97/39 | Sof11 + WD40 | 482/445 | [49] |
|  |  |  |  | PF3D7_1237600 | rRNA processing WD-repeat protein, putative | NP_008993.1 | Periodic tryptophan protein 1 homolog (PWP1) | 2e-27 | 73/28 | WD40 | 502/501 | [3,59,60] |
|  |  |  |  | PF3D7_1405800 | Large subunit rRNA processing protein, putative | NP_056016.1 | Ribosome biogenesis protein BOP1 | 8e-32 | 53/30 | BOP1NT + WD40 | 966/746 | [61] |
|  |  |  |  | PF3D7_1333600 | U3 snoRNA-associated small subunit rRNA processing associated protein, putative | NP_116219.1 | Cirhin or UTP4 | 0.005 | 24/20 | Collagen^Pf^ + Transmembrane ^Pf^ + WD40 + Coiled coil^Pf^ | 1335/686 | [62] |
|  |  |  |  | PF3D7_0801500 | Conserved Plasmodium protein, unknown function | NP_079170.2 | nucleolar protein10 (NOL10) | 9e-90 | 97/34 | WD40 + NUC153^Hs^ | 549/688 | [49] |
|  |  |  |  | PF3D7_1226700 | Conserved Plasmodium protein, unknown function | NP_004695.1 | RNA U3 small nucleolar interacting protein 2 (RRP9) | 1e-27 | 48/22 | WD40 | 636/475 | [63,64] |
|  |  |  |  | PF3D7_0630500 | Microtubule-associated protein ytm1 homologue, putative | NP_060726.3  [*S. cerevisiae* - YOR272W] | Ribosome biogenesis protein WDR12  [Ribosome biogenesis protein YTM1] | 0.001  [6e-18] | 15/33  [79/24] | NLE + WD40 | 498/423  [498/460] | [3,61,65] |
|  |  |  |  | PF3D7_0526300 | Nucleolar Jumonji domain interacting protein, putative | NP_060176.2 | WDR55 | 2e-23 | 98/22 | WD40 | 323/383 | [66] |
|  |  | | | PF3D7_0816000 | Nucleolar preribosomal assembly protein, putative | NP_113673.2 | Glutamate-rich WD repeat-containing protein1 | 4e-44 | 80/36 | CAF1C_H4-bd + WD40 | 491/446 | [3,49,67] |
|  |  | | | PF3D7_1146000 | Nucleolar preribosomal assembly protein, putative | NP_060566.2 | Notchless protein homolog 1 | 1e-46 | 97/29 | WD40 + NLE | 645/485 | [3,49] |
|  | **b) pre-mRNA processing** | | | PF3D7_0308600 | Pre-mRNA-processing factor 19, putative (PRPF19) | NP_055317.1 | Pre-mRNA-processing factor 19 | 8e-85 | 100/33 | Prp19 + Ubox + WD40 | 532/504 | [3,49,68-70] |
|  |  |  |  | PF3D7_1241100 | Conserved Plasmodium protein, unknown function | NP_060853.3 | Pre-mRNA 3' end processing protein WDR33 | 1e-39 | 95/27 | WD40 + collagen^Hs^ | 468/1336 | [71,72] |
|  |  |  |  | PF3D7_0620500 | Cleavage stimulation factor subunit 1-like protein, putative | NP_001315.1 | Cleavage stimulation factor subunit 1 | 9e-23 | 36/27 | WD40 | 650/431 | [73,74] |
|  |  |  |  | PF3D7_1220100 | Pre-mRNA splicing factor, putative | NP_056975.1 | Pre-mRNA-processing factor 17 | 2e-83 | 85/35 | WD40 | 618/579 | [75,76] |
|  |  |  |  | PF3D7_0822800 | U5 snrnp-specific protein, putative | NP_004805.2 | U5 small nuclear ribonucleoprotein 40 kDa protein | 3e-57 | 100/35 | WD40 | 324/357 | [77,78] |
|  |  |  |  | PF3D7_1343900 | U4/U6 small nuclear ribonucleoprotein PRP4, putative | NP_004688.2 | U4/U6 small nuclear ribonucleoprotein Prp4 | 2e-35 | 81/43 | PRP4 + WD40 | 648/522 | [78] |
|  |  |  |  | PF3D7_0302000 | Golgi organization and biogenesis factor, putative | NP_002660.1 | Pleiotropic regulator 1 | 3e-132 | 54/52 | WD40 | 600/514 | [79] |
|  |  |  |  | PF3D7_1311200 | Alternative splicing regulator, putative | NP_060695.2 | WD40 repeat-containing protein SMU1 | 2e-130 | 98/37 | WD40 + LisH + CTLH^Hs^ | 527/513 |  |
| **Translation initiation factor/Post-translational modifications/Protein folding** | | | | PF3D7_0716800 | Eukaryotic translation initiation factor 3 37.28 kDa subunit, putative | NP_003748.1 | Eukaryotic translation initiation factor 3 subunit | 9e-51 | 98/32 | WD40 | 327/325 | [3,80] |
|  |  |  |  | PF3D7_0608000 | Conserved Plasmodium protein, unknown function | NP_620133.1 | WD repeat-containing protein 85 (WDR85)  (Protein modification- dipthamide biosynthetic pathway) | 2e-16 | 72/26 | WD40 | 345/452 | [81] |
|  |  |  |  | PF3D7_0510200 | Peptidyl-prolyl cis-trans isomerase (CYP87)  (Cyclophilin type molecular chaperone) | NP_056157.1 | Peptidylprolyl isomerase domain and WD repeat-containing protein 1  (Identified in spliceosome C complex) | 3e-142 | 86/39 | Pro_isomerase + WD40 | 747/646 | [77,82] |
| **Cytoskeletal regulatory proteins** | | **a) Dynein- intermediate chain** | | PF3D7_0922000 | Dynein intermediate chain 2, ciliary | NP_036276.1 | Dynein intermediate chain 1, axonemal | 3e-42 | 64/31 | WD40 | 820/699 | [83-85] |
|  |  |  |  | PF3D7_1020300 | Cytoplasmic dynein intermediate chain, putative | NP_001369.1 | Cytoplasmic dynein 1 intermediate chain 2 | 6e-85 | 55/37 | WD40 + Dynein IC2^Hs^ | 769/638 | [83] |
|  |  |  |  | PF3D7_1037800 | Dynein intermediate chain, putative | NP_660155.1 | WDR63/ Testis development protein NYD-SP29 | 1e-16 | 72/22 | WD40 + Transmembrane^Pf^ | 944/891 | [86] |
|  |  |  |  | PF3D7_1426300 | Dynein-associated protein, putative | NP_075462.2 | Dynein intermediate chain 2, axonemal | 4e-62 | 95/28 | WD40 | 605/605 | [87,88] |
|  |  | **b) Myosin** | | PF3D7_1329100 | Myosin C (MyoC) or Myosin F ^89^ | _ | _ | _ | _ | WD40 + IQ + Myosin motor domain + Coiled coil | 2160/- | [89] |
|  |  | **c) Actin related proteins** | | PF3D7_1118800 | Conserved Plasmodium protein, unknown function | NP_006400.2 | Actin-related protein 2/3 complex subunit 1A | 1e-06 | 94/20 | WD40 + ARP2/3_su1^Hs^ | 391/370 | [90] |
|  |  | **d) Microtubule associated protein (flagellum axoneme)** | | PF3D7_0510800 | Conserved Plasmodium protein, unknown function | NP_078808.3 | Sperm associated antigen 16 protein (SPAG16) or or Pf20 protein homolog | 3e-84 | 80/31 | WD40 | 642/631 | [91] |
|  |  | **e) Basal body, axoneme** | | PF3D7_1348700 | Conserved Plasmodium protein, unknown function | NP_659491.4 | WD repeat-containing protein 16 (WDR16) | 8e-48 | 87/21 | WD40 | 715/620 | [92,93] |
|  |  | **f) Flagellar protein** | | PF3D7_1406500 | Conserved Plasmodium protein, unknown function | NP_689711.1 | WD repeat-containing protein 65 (WDR65)  (Trypanosome homolog - DIGIT (Tb927.8.4870) ) | 2e-34 | 64/28 | WD40 | 1527/1250 | [94,95] |
| **Others** | | | | PF3D7_1315400 | Zinc finger (CCCH type) protein, putative | _ | _ | _ | _ | 1ZF_C3H1 + WD40 | 419/- | [3] |
|  |  |  |  | PF3D7_1221600 | Conserved Plasmodium protein, unknown function | NP_003301.1 | Tumor-suppressing STF cDNA 1 protein (TSSC1) | 8e-26 | 53/32 | WD40 | 458/387 | [96] |
|  |  |  |  | PF3D7_1209400 | Conserved Plasmodium protein, unknown function OR cytosolic iron-sulfur protein assembly protein 1, putative | NP_004795.1 | Probable cytosolic iron-sulfur protein assembly protein CIAO1(Fe/S protein biogenesis) | 1e-17 | 44/31 | WD40 | 792/339 | [97-,99] |
|  |  |  |  | PF3D7_0905600 | Conserved Plasmodium protein, unknown function | NP_653269.3 | WD repeat-containing protein 66 isoform 1 (Calcium ion binding) | 2e-11 | 52/17 | WD40 | 1220/1149 | [100,101] |
| ***Pf*WDR proteins, unknown function (with no/doubtful human homologs)** | | | | PF3D7_0525500 | Conserved Plasmodium protein, unknown function |  |  |  |  | WD40 |  |  |
|  |  |  |  | PF3D7_1104500 | Conserved Plasmodium protein, unknown function | NP_758440.1 | POC1 centriolar protein homolog B | 2e-06 | 47/37 | WD40 + coiled coil^Hs^ | 644/478 | [102] |
|  |  |  |  | PF3D7_1138800 | Conserved Plasmodium protein, unknown function |  |  |  |  | WD40 |  | [3] |
|  |  |  |  | PF3D7_1121400 | WD domain, G-beta repeat-containing protein |  |  |  |  | WD40 |  |  |
|  |  |  |  | PF3D7_1029200 | Conserved Plasmodium protein, unknown function |  |  |  |  | WD40 |  |  |
|  |  |  |  | PF3D7_1230500 | WD-repeat protein, putative |  |  |  |  | WD40 |  |  |
|  |  |  |  | PF3D7_1443400 | Conserved Plasmodium protein, unknown function |  |  |  |  | WD40 |  |  |
|  |  |  |  | PF3D7_1212300 | Conserved Plasmodium protein, unknown function | NP_079039.3 | WDR78 | 1e-19 | 32/33 | WD40 | 1251/848 |  |
|  |  |  |  | PF3D7_1228800 | conserved Plasmodium protein, unknown function |  |  |  |  | WD40 + Transmembrane |  | [3] |
|  |  |  |  | PF3D7_1409000 | Conserved Plasmodium protein, unknown function |  |  |  |  | WD40 |  |  |
|  |  |  |  | PF3D7_0321800 | Conserved Plasmodium protein, unknown function |  |  |  |  | WD40 |  |  |
|  |  |  |  | PF3D7_1410300 | Conserved Plasmodium protein, unknown function |  |  |  |  | WD40 + PbH1 + Coiled coil |  |  |
|  |  |  |  | PF3D7_1459200 | Conserved Plasmodium protein, unknown function |  |  |  |  | WD40 |  |  |
|  |  |  |  | PF3D7_1004200 | Conserved Plasmodium membrane protein, unknown function |  |  |  |  | WD40 + Transmembrane |  |  |
|  |  |  |  | PF3D7_1004100 | Hypothetical protein |  |  |  |  | WD40 |  |  |
|  |  |  |  | PF3D7_1326100 | Conserved Plasmodium protein, unknown function | NP_079498.2 | WD repeat-containing protein 82 | 2e-07 | 61/20 | WD40 | 336/313 |  |

**References**

1. Verreault A, Kaufman PD, Kobayashi R, Stillman B (1996) Nucleosome assembly by a complex of CAF-1 and acetylated histones H3/H4. Cell 87: 95-104.
2. Zhang Q, Vo N, Goodman RH (2000) Histone binding protein RbAp48 interacts with a complex of CREB binding protein and phosphorylated CREB. Mol Cell Biol 20: 4970-4978.
3. Oehring SC, Woodcroft BJ, Moes S, Wetzel J, Dietz O, Pulfer A, et al. (2012) Organellar proteomics reveals hundreds of novel nuclear proteins in the malaria parasite Plasmodium falciparum. Genome Biol 13: R108.
4. Das C, Tyler JK, Churchill ME (2010) The histone shuffle: histone chaperones in an energetic dance. Trends Biochem Sci 35: 476-489.
5. Wu M, Wang PF, Lee JS, Martin-Brown S, Florens L, Washburn M, et al. (2008) Molecular regulation of H3K4 trimethylation by Wdr82, a component of human Set1/COMPASS. Mol Cell Biol 28: 7337-7344.
6. Lee JH, You J, Dobrota E, Skalnik DG (2010) Identification and characterization of a novel human PP1 phosphatase complex. J Biol Chem 285: 24466-24476.
7. Bi Y, Lv Z, Wang Y, Hai T, Huo R, Zhou Z, et al. (2011) WDR82, a key epigenetics-related factor, plays a crucial role in normal early embryonic development in mice. Biol Reprod 84: 756-764.
8. **Enninga J, Levay A, Fontoura BM (2003)** Sec13 shuttles between the nucleus and the cytoplasm and stably interacts with Nup96 at the nuclear pore complex. Mol Cell Biol 23: 7271-7284.
9. Dahan-Pasternak N, Nasereddin A, Kolevzon N, Pe'er M, Wong W, Shinder V,  et al. (2013) PfSec13 is an unusual chromatin-associated nucleoporin of Plasmodium falciparum that is essential for parasite proliferation in human erythrocytes. J Cell Sci 126: 3055-3069.
10. Adisa A, Albano FR, Reeder J, Foley M, Tilley L (2001) Evidence for a role for a *Plasmodium falciparum* homologue of Sec31p in the export of proteins to the surface of malaria parasite-infected erythrocytes. J Cell Sci 114: 3377-3386.
11. Adisa A, Frankland S, Rug M, Jackson K, Maier AG, Walsh P, et al. (2007) Re-assessing the locations of components of the classical vesicle-mediated trafficking machinery in transfected Plasmodium falciparum. Int J Parasitol 37: 1127-1141.
12. Jensen D, Schekman R (2011) COPII-mediated vesicle formation at a glance. J Cell Sci 124: 1-4.
13. Lee MCS, Moura PA, Miller EA, Fidock DA (2008) *Plasmodium falciparum* Sec24 marks transitional ER that exports a model cargo via a diacidic motif. Mol Microbiol 68: 1535-1546.
14. Chow VTK, Quek HH (1996) HEP-COP, a novel human gene whose product is highly homologous to the alpha-subunit of the yeast coatomer protein complex. Gene 169: 223-227.
15. Harrison-Lavoie KJ, Lewis VA, Hynes GM, Collison KS, Nutland E (1993) A 102 kDa subunit of Golgi-associated particle has homology to beta subunits of trimeric G proteins. EMBO J 12: 2847-2853.
16. Tardieuxa I, Liu X, Poupel O, Parzy D, Dehoux P, Langsley G (1998) A *Plasmodium falciparum* novel gene encoding a coronin-like protein which associates with actin ¢laments. FEBS Letters 441: 251-256.
17. Proikas-Cezanne T, Waddell S, Gaugel A, Frickey T, Lupas A, Nordheim A (2004) WIPI-1alpha (WIPI49), a member of the novel 7-bladed WIPI protein family, is aberrantly expressed in human cancer and is linked to starvation-induced autophagy. Oncogene 23: 9314-9325.
18. Duszenko M, Ginger ML, Brennand A, Gualdrón-López M, Colombo MI, Coombs GH, Coppens I, et al. (2012) Autophagy in protists. Autophagy 7: 127-158.
19. Gaugel A, Bakula D, Hoffmann A, Proikas-Cezanne T (2012) Defining regulatory and phosphoinositidebinding sites in the human WIPI-1 β-propeller responsible for autophagosomal membrane localization downstream of mTORC1 inhibition. J Mol Signal 7: 16.
20. Cervantes S, Bunnik EM, Saraf A, Conner CM, Escalante A, Sardiu ME, et al. (2014) The multifunctional autophagy pathway in the human malaria parasite, *Plasmodium falciparum*. Autophagy 10: 80-92.
21. Liliental J, Chang DD (1998) Rack1, a receptor for activated protein kinase C, interacts with integrin-β subunit. J Biol Chem 273: 2379-2383.
22. Sartorelloa R, Amayab MJ, Nathansonb MH, Garciac CRS (2009) The *Plasmodium* receptor for activated C kinase protein inhibits Ca2+ signaling in mammalian cells. Biochem Biophys Res Commun 389: 586-592.
23. Adams DA, Ron D, Kiely PA (2011) RACK1, A multifaceted scaffolding protein: Structure and function. Cell Commun Signal 9: 22.
24. Vomastek T, Schaeffer HJ, Tarcsafalvi A, Smolkin ME, Bissonette EA, Weber MJ (2004) Modular construction of a signaling scaffold: MORG1 interacts with components of the ERK cascade and links ERK signaling to specific agonists. Proc Natl Acad Sci USA 101: 6981-6986.
25. Hayase J, Kamakura S, Iwakiri Y, Yamaguchi Y, Izaki T, Ito T, et al. (2004) The WD40 protein Morg1 facilitates Par6–aPKC binding to Crb3 for apical identity in epithelial cells. J Cell Biol 200: 635-650.
26. Zhu Y, Wang Y, Xia C, Li D, Li Y, Zeng W, et al. (2004) WDR26: A novel Gβ‐like protein, suppresses MAPK signaling pathway. J cell Biochem 93: 579-587.
27. Sun Z, Smrcka AV, Chen S (2013) WDR26 functions as a scaffolding protein to promote Gβγ-mediated phospholipase C β2 (PLCβ2) activation in leukocytes. J Biol Chem 288: 16715-16725.
28. Fang G, Yu H, Kirschner MW (1998) Direct binding of CDC20 protein family members activates the anaphase-promoting complex in mitosis and G1. Mol Cell 2: 163-171.
29. Shirayama M, Zachariae W, Ciosk R, Nasmyth K (1998) The Polo-like kinase Cdc5p and the WD-repeat protein Cdc20p/fizzy are regulators and substrates of the anaphase promoting complex in *Saccharomyces cerevisiae*. EMBO J 17: 1336-1349.
30. Zhou Y, Ching YP, Chun AC, Jin DY (2003) Differential expression, localization and activity of two alternatively spliced isoforms of human APC regulator CDH1. Biochem J 374: 349-358.
31. Huang H, Summers MK, Pham V, Lill JR, Liu J, Lee G, et al. (2011) Deubiquitinase USP37 is activated by CDK2 to antagonize APC (CDH1) and promote S-phase entry.  Mol Cell 42: 511-523.
32. Guttery DS, Ferguson DJP, Poulin B, Xu Z, Straschil U, Klop O, et al. (2012) A putative homologue of CDC20/CDH1 in the malaria parasite is essential for male gamete development. PLoS Pathog 8: e1002554.
33. Koshizuka Y, Ikegawa S, Sano M, Nakamura K, Nakamura Y (2001) Isolation, characterization, and mapping of the mouse and human WDR8 genes, members of a novel WD-repeat gene family. [*Genomics*](http://www.ncbi.nlm.nih.gov/pubmed?cmd=search&term=11401440&dopt=b) 72: 252-259.
34. Jakobsen L, Vanselow K, Skogs M, Toyoda Y, Lundberg E, Poser I, et al. (2011) Novel asymmetrically localizing components of human centrosomes identified by complementary proteomics methods. EMBO J 30: 1520-1535.
35. Shen KF, Osmani SA (2013) Regulation of mitosis by the NIMA kinase involves TINA and its newly discovered partner, An-WDR8, at spindle pole bodies. Mol Biol Cell 24: 3842-3856.
36. Rohrschneider MR, Nance J (2009) Polarity and cell fate specification in the control of *Caenorhabditis elegans* gastrulation. Dev Dyn 238: 789-796.
37. Venteicher AS, Abreu EB, Meng Z, McCann KE, Terns RM, Veenstra TD, et al. (2009) A human telomerase holoenzyme protein required for Cajal body localization and telomere synthesis. Science 323: 644-648.
38. Mahmoudi S, Henriksson S, Corcoran M, Méndez-Vidal C, Wiman KG, Farnebo M (2009) Wrap53, a natural p53 antisense transcript required for p53 induction upon DNA damage. Mol Cell 33: 462-471.
39. Saldaña-Meyer R, Recillas-Targa F (2011) Transcriptional and epigenetic regulation of the p53 tumor suppressor gene. Epigenetics 6: 1068-1077.
40. Saeki M, Irie Y, Ni L, Yoshida M, Itsuki Y, Kamisaki Y (2006) Monad, a WD40 repeat protein, promotes apoptosis induced by TNF-α. Biochem Biophys Res Commun 342: 568-572.
41. Xu C, Min J (2011) Structure and function of WD40 domain proteins. Protein Cell 2: 202-214.
42. Saeki M, Egusa H, Kamano Y, Kakihara Y, Houry WA, Yatani H, et al. (2013) Exosome-bound WD repeat protein Monad inhibits breast cancer cell invasion by degrading amphiregulin mRNA. PloS One 8: e67326.
43. Adam-Klages S, Adam D, Wiegmann K, Struve S, Kolanus W, Schneider-Mergener J, et al. (1996) FAN, a novel WD-repeat protein, couples the p55 TNF-receptor to neutral sphingomyelinase. Cell 86: 937-947.
44. Behrends C, Sowa ME, Gygi SP, Harper JW (2010) Network organization of the human autophagy system. Nature 466: 68-76.
45. Chopra AK, Ribardo DA, Wood TG, Prusak DJ, Xu XJ, Peterson JW (1999) Molecular characterization of cDNA for phospholipase A2-activating protein. Biochim Biophys Acta 1444: 125-130.
46. Fu QS, Zhou CJ, Gao HC, Jiang YJ, Zhou ZR, Hong J (2009) Structural basis for ubiquitin recognition by a novel domain from human phospholipase A2-activating protein. J Biol Chem 284: 19043-1952.
47. Qiu L, Pashkova N, Walker JR, Winistorfer S, Allali-Hassani A, Akutsu M, et al. (2010) Structure and function of the PLAA/Ufd3-p97/Cdc48 complex. J Biol Chem 285: 365-372.
48. Angers S, Li T, Yi X, MacCoss MJ, Moon RT, Zheng N (2006) Molecular architecture and assembly of the DDB1–CUL4A ubiquitin ligase machinery. Nature 443: 590-593.
49. Jin J, Arias EE, Chen J, Harper JW, Walter JC (2006) A family of diverse Cul4-Ddb1-interacting proteins includes Cdt2, which is required for S phase destruction of the replication factor Cdt1. Mol Cell 23: 709-721.
50. Scherl A, Couté Y, Déon C, Callé A, Kindbeiter K, Sanchez JC,  et al. (2002) Functional proteomic analysis of human nucleolus. Mol Biol Cell 13: 4100-4109.
51. Yamakawa K, Gao DQ, Korenberg JR (1996) A periodic tryptophan protein 2 gene homologue (PWP2H) in the candidate region of progressive myoclonus epilepsy on 21q22.3. Cytogenet Cell Genet 74: 140-145.
52. Claudio JO, Liew CC, Ma J, Heng HH, Stewart AK, Hawley RG (1999) Cloning and expression analysis of a novel WD repeat gene, WDR3, mapping to 1p12-p13. [Genomics](http://www.ncbi.nlm.nih.gov/pubmed/10395803) 59: 85-89.
53. Weinstat-Saslow DL, Germino GG, Somlo S, Reeders ST (1993) A transducin-like gene maps to the autosomal dominant polycystic kidney disease gene region. Genomics 18: 709-711.
54. Takezawa S, Yokoyama A, Okada M, Fujiki R, Iriyama A, Yanagi Y, et al. (2007) A cell cycle-dependent co-repressor mediates photoreceptor cell-specific nuclear receptor function. EMBO J 26: 764-774.
55. Hauser MA, Allingham RR, Linkroum K, Wang J, LaRocque-Abramson K, Figueiredo D, et al. (2006) Distribution of WDR36 DNA sequence variants in patients with primary open-angle glaucoma*.* Invest Ophthalmol Vis Sci 47: 2542-2546.
56. Herberg JA, Beck S, Trowsdale J (1998) TAPASIN, DAXX, RGL2, HKE2 and four new genes (BING 1, 3 to 5) form a dense cluster at the centromeric end of the MHC. J Mol Biol 277: 839-857.
57. Pasaje CF, Bae JS, Park BL, Cheong HS, Kim JH, Uh ST,  et al. (2012) WDR46 is a genetic risk factor for aspirin-exacerbated respiratory disease in a Korean population. Allergy Asthma Immunol Res 4: 199-205.
58. Hirai Y, Louvet E, Oda T, Kumeta M, Watanabe Y, Horigome T, et al. (2013) Nucleolar scaffold protein, WDR46, determines the granular compartmental localization of nucleolin and DDX21. Genes Cells 18: 780-797.
59. Honoré B, Leffers H, Madsen P (1994) Cloning of a cDNA encoding a novel human nuclear phosphoprotein belonging to the WD-40 family. Gene 151: 291-296.
60. Suka N, Nakashima E, Shinmyozu K, Hidaka M, Jingami H (2006) The WD40-repeat protein Pwp1p associates in vivo with 25S ribosomal chromatin in a histone H4 tail-dependent manner. Nucleic Acids Res 34: 3555-3567.
61. Rohrmoser M, Hölzel M, Grimm T, Malamoussi A, Harasim T, Orban M, et al. (2007) Interdependence of Pes1, Bop1, and WDR12 controls nucleolar localization and assembly of the PeBoW complex required for maturation of the 60S ribosomal subunit. Mol Cell biol 27: 3682-3694.
62. Bréhélin L, Florent I, Gascuel O, Maréchal E (2010) Assessing functional annotation transfers with inter-species conserved coexpression: application to *Plasmodium falciparum*. BMC Genomics 11: 35.
63. Lukowiak AA, Granneman S, Mattox SA, Speckmann WA, Jones K, Pluk H, et al. (2000) Interaction of the U3-55k protein with U3 snoRNA is mediated by the box B/C motif of U3 and the WD repeats of U3-55k. Nucleic acids res 28: 3462-3471.
64. Granneman S, Pruijn GJM, Horstman W, van Venrooij WJ, Lührmann R, Watkins NJ, et al. (2002) The hU3-55K protein requires 15.5K binding to the box B/C motif as well as flanking RNA elements for its association with the U3 small nucleolar RNA in vitro. J Biol Chem 277: 48490-48500.
65. Hölzel M, Rohrmoser M, Schlee M, Grimm T, Harasim T, Malamoussi A, et al. (2005) Mammalian WDR12 is a novel member of the Pes1-Bop1 complex and is required for ribosome biogenesis and cell proliferation. J Cell Biol 170: 367-378.
66. Iwanami N, Higuchi T, Sasano Y, Fujiwara T, Hoa VQ, Okada M,  et al. (2008) WDR55 is a nucleolar modulator of ribosomal RNA synthesis, cell cycle progression, and teleost organ development. PLoS Genet 4: e1000171.
67. Cloutier P, Lavallée-Adam M, Faubert D, Blanchette M, Coulombe B (2013) A newly uncovered group of distantly related lysine methyltransferases preferentially interact with molecular chaperones to regulate their activity. PLoS Genet 9: e1003210.
68. Gotzmann J, Gerner C, Meissner M, Holzmann K, Grimm R, Mikulits W , et al. (2000) hNMP 200: a novel human common nuclear matrix protein combining structural and regulatory functions. Exp Cell 2000, 261: 166-179.
69. Grillari J, Ajuh P, Stadler G, Löscher M, Voglauer R, Ernst W, et al. (2005) SNEV is an evolutionarily conserved splicing factor whose oligomerization is necessary for spliceosome assembly. Nucleic Acids Res 33: 6868-6883.
70. Grote M, Wolf E, Will CL, Lemm I, Agafonov DE, Schomburg A , et al. (2010) Molecular architecture of the human Prp19/CDC5L complex. Mol Cell Biol 30: 2105-2119.
71. Ito S, Sakai A, Nomura T, Miki Y, Ouchida M, Sasaki J, et al. (2001) A novel WD40 repeat protein, WDC146, highly expressed during spermatogenesis in a stage-specific manner. Biochem Biophys Res Commun 280: 656-663.
72. Shi Y, Stefan CJ, Rue SM, Teis D, Emr SD (2011) Two novel WD40 domain–containing proteins, Ere1 and Ere2, function in the retromer-mediated endosomal recycling pathway. Mol Biol Cell 22: 4093-4107.
73. Takagaki Y, Manley JL (1992) A human polyadenylation factor is a G protein beta-subunit homologue. J Biol Chem 267: 23471-23474.
74. Kleiman FE, Manley JL (1999) Functional interaction of BRCA1-associated BARD1 with polyadenylation factor CstF-50. Science 285: 1576-1579.
75. Lindsey LA, Garcia-Blanco MA (1998) Functional conservation of the human homolog of the yeast pre-mRNA splicing factor Prp17p. J Biol Chem 273: 32771-32775.
76. Yehuda SB, Dixi [I](http://www.ncbi.nlm.nih.gov/pubmed?term=Dix I%5BAuthor%5D&cauthor=true&cauthor_uid=9769104), Russell CS, Levy S, Beggs JD, Kupiec M (1998) Identification and functional analysis of hPRP17, the human homologue of the PRP17/CDC40 yeast gene involved in splicing and cell cycle control. RNA 4: 1304-1312.
77. Jurica MS, Licklider LJ, Gygi SP, Grigorieff N, Moore MJ (2002) Purification and characterization of native spliceosomes suitable for three-dimensional structural analysis. RNA 8: 426-439.
78. Liu S, Rauhut R, Vornlocher HP, Luhrmann R (2006) The network of protein–protein interactions within the human U4/U6.U5 tri-snRNP. RNA 12: 1418-1430.
79. Ajuh P, Sleeman J, Chusainow J, Lamond AI (2001) A direct interaction between the carboxyl-terminal region of CDC5L and the WD40 domain of PLRG1 is essential for pre-mRNA splicing. J Biol Chem 276: 42370-42381.
80. Siridechadilok B, Fraser CS, Hall RJ, Doudna JA, Nogales E (2005) Structural roles for human translation factor eIF3 in initiation of protein synthesis. Science 310: 1513-1515.
81. Carette JE, Guimaraes CP, Varadarajan M, Park AS, Wuethrich I, Godarova A et al. (2009) Haploid genetic screens in human cells identify host factors used by pathogens. Science 326: 1231-1235.
82. Marin-Menendez A, Bell A (2010) Identification and characterization of novel *Plasmodium falciparum* cyclophilins and their roles in the antimalarial actions of cyclosporine A and derivatives. Malaria J 9:O3.
83. Vaughan KT, Vallee RB (1995) Cytoplasmic dynein binds dynactin through a direct interaction between the intermediate chains and p150 glued. J Cell Biol 131: 1507-1516.
84. Pennarun G, Escudier E, Chapelin C, Bridoux AM, Cacheux V, Roger G , et al. (1999) Loss-of-function mutations in a human gene related to *Chlamydomonas reinhardtii* dynein IC78 result in primary ciliary dyskinesia. Am J Hum Genet 65: 1508–1519.
85. Guichard C, Harricane MC, Lafitte JJ, Godard P, Zaegel M, Tack V, et al. (2001) Axonemal dynein intermediate-chain gene (DNAI1) mutations result in situs inversus and primary ciliary dyskinesia (Kartagener syndrome). Am J Hum Genet 68: 1030-1035.
86. King SM (2012) Composition and assembly of axonemal dyneins. In: King SM, Waltham, MA, editors. Dyneins: Structure, Biology and Disease. Elsevier. pp. 209-243.
87. Pennarun G, Chapelin C, Escudier E, Bridoux AM, Dastot F, Cacheux V, et al. (2000) The human dynein intermediate chain 2 gene (DNAI2): cloning, mapping, expression pattern, and evaluation as a candidate for primary ciliary dyskinesia. Hum Genet 107: 642-649.
88. Loges NT, Olbrich H, Fenske L, Mussaffi H, Horvath J, Fliegauf M, et al. (2008) DNAI2 mutations cause primary ciliary dyskinesia with defects in the outer dynein arm. Am J Hum Genet 83: 547-558.
89. Foth BJ, Goedecke MC, Soldati D (2006) New insights into myosin evolution and classification. Proc Natl Acad Sci USA 103: 3681-3686.
90. Gordon JL, Sibley LD (2005) Comparative genome analysis reveals a conserved family of actin-like proteins in apicomplexan parasites. BMC Genomics 6: 179.
91. Zhang Z, Sapiro R, Kapfhamer D, Bucan M, Bray J, Chennathukuzhi V, et al. (2002) A sperm-associated WD repeat protein orthologous to *Chlamydomonas* PF20 associates with Spag6, the mammalian orthologue of *Chlamydomonas* PF16. Mol Cell Biol 22: 7993-8004.
92. Silva FP, Hamamoto R, Nakamura Y, Furukawa Y (2005) WDRPUH, a novel WD-repeat-containing protein, is highly expressed in human hepatocellular carcinoma and involved in cell proliferation. Neoplasia 7: 348-355.
93. Hodges ME, Scheumann N, Wickstead B, Langdale JA, Gull K (2010) Reconstructing the evolutionary history of the centriole from protein components. J Cell Sci 123: 1407-1413.
94. Broadhead R, Dawe HR, Farr H, Griffiths S, Hart SR, Portman N, et al. (2006) Flagellar motility is required for the viability of the bloodstream trypanosome. Nature 440: 224-227.
95. Rorick NK, Kinoshita A, Weirather JL, Peyrard-Janvid M, de Lima RLLF, Dunnwald M, et al. (2011) Genomic strategy identifies a missense mutation in WD-repeat domain 65 (WDR65) in an individual with Van der Woude syndrome. Am J Med Genet Part A 155A: 1314-1321.
96. Hu RJ, Lee MP, Connors TD, Johnson LA, Burn TC, Su K, et al. (1997) A 2.5-Mb transcript map of a tumor-suppressing subchromosomal transferable fragment from 11p15. 5, and isolation and sequence analysis of three novel genes. Genomics 46: 9-17.
97. Johnstone RW, Wang J, Tommerup N, Vissing H, Roberts T, Shi Y (1998) Ciao 1 is a novel WD40 protein that interacts with the tumor suppressor protein WT1. J Biol Chem 273: 10880-10887.
98. Srinivasan V, Netz DJ, Webert H, Mascarenhas J, Pierik AJ, Michel H,  et al. (2007) Structure of the yeast WD40 domain protein Cia1, a component acting late in iron-sulfur protein biogenesis. [Structure](http://www.ncbi.nlm.nih.gov/pubmed/17937914)15: 1246-1257.
99. Ito S, Tan LJ, Andoh D, Narita T, Seki M, Hirano Y,  et al. (2010) MMXD, a TFIIH-independent XPD-MMS19 protein complex involved in chromosome segregation. Mol Cell 39: 632-40.
100. Ochoa A, Llinás M, Singh M (2011) Using context to improve protein domain identification. BMC Bioinformatics 12: 90.
101. Wang Q, Ma C, Kemmner W (2013) Wdr66 is a novel marker for risk stratification and involved in epithelial-mesenchymal transition of esophageal squamous cell carcinoma. BMC cancer 13: 137.
102. Woodland HR, Fry AM (2008) Pix Proteins and the Evolution of Centrioles. PLoS One 3: e3778.
