# Supplementary material for: Genome-Wide Collation of the Plasmodium falciparum WDR Protein Superfamily Reveals Malarial Parasite-Specific Features
Source: PLoS One. 2015 Jun 4;10(6):e0128507. doi: 10.1371/journal.pone.0128507 (PMC4456382; doi:10.1371/journal.pone.0128507)
Supplement: S6 Table — (DOCX) [file pone.0128507.s011.docx]

**Table S6. Characteristics of the homology modeled 3D structures of 23 *Pf*WDR proteins.**

| **Sr. No.** | **Gene ID** | **Protein Description** | **Percentage of residues modeled** | **Percentage of residues in allowed regions** | **Percentage of residues in outlier region** | **QMEAN score** | **Domain composition** |
| --- | --- | --- | --- | --- | --- | --- | --- |
|  | PF3D7_1012900 | autophagy-related protein 18, putative | 372/380(98%) | 91.5% | 8.5% | 0.328 | 2 WD40 |
|  | PF3D7_1363400 | polyubiquitin binding protein, putative | 787/905(87%) | 92.5% | 7.5% | 0.356 | 7 WD40 + PFU +PUL |
|  | PF3D7_1146000 | nucleolar preribosomal assembly protein, putative | 632/645(98%) | 88.8% | 11.2% | 0.444 | 8 WD40 + 1 NLE |
|  | PF3D7_1347000 | G-beta repeat protein , putative | 356/363(98%) | 98.9% | 1.1% | 0.606 | 4 WD40 |
|  | PF3D7_0822800 | U5 snrnp-specific protein, putative | 324/324(100%) | 94.5% | 5.6% | 0.434 | 7 WD40 |
|  | PF3D7_0526300 | nucleolar Jumonji domain interacting protein, putative | 316/323 (98%) | 84.6% | 15.5% | 0.428 | 5 WD40 |
|  | PF3D7_0826700 | receptor for activated c kinase (RACK) | 313/323 (97%) | 98.1% | 1.9% | 0.747 | 7 WD40 |
|  | PF3D7_0716800 | eukaryotic translation initiation factor 3 37.28 kDa subunit, putative | 327/327 (100%) | 92% | 8.0% | 0.435 | 6 WD40 |
|  | PF3D7_0803300 | mitogen-activated protein kinase organizer 1, putative (*Pf*MORG1) | 357/365 (98%) | 95.9% | 4.1% | 0.535 | 6 WD40 |
|  | PF3D7_1026400 | cell division cycle protein 20 homolog, putative | 524/603 (87%) | 94.9% | 5.2% | 0.411 | 7 WD40 |
|  | PF3D7_0308600 | pre-mRNA-processing factor 19, putative (PRPF19) | 494/532 (93%) | 93.4% | 6.6% | 0.436 | 6 WD40 + 1 Ubox + 1Prp19 |
|  | PF3D7_0608000 | conserved Plasmodium protein, putative | 310/345 (90%) | 93.2% | 6.7% | 0.430 | 3 WD40 |
|  | PF3D7_1243800 | microtubule associated katanin, putative | 362/370 (98%) | 88.9% | 11.1% | 0.421 | 5 WD40 |
|  | PF3D7_1221600 | conserved Plasmodium protein, Putative | 393/458 (86%) | 94.8% | 5.3% | 0.269 | 3 WD40 |
|  | PF3D7_1315400 | zinc finger (CCCH type) protein, putative | 389/419 (93%) | 89.2% | 10.8% | 0.304 | 1 WD40 + 1 ZF_C3H1 |
|  | PF3D7_0110700 | chromatin assembly factor 1 protein WD40 domain, putative | 374/446 (84%) | 98.4% | 1.6% | 0.582 | 6 WD40 + 1 [CAF1C_H4-bd](http://pfam.sanger.ac.uk/family/PF12265.3) |
|  | PF3D7_1433300 | chromatin assembly factor 1 P55 subunit, putative | 376/428 (88%) | 96.3% | 3.8% | 0.528 | 6 WD40 + 1 [CAF1C_H4-bd](http://pfam.sanger.ac.uk/family/PF12265.3) |
|  | PF3D7_1105200 | conserved Plasmodium protein, putative | 458/463 (99%) | 95.5% | 4.6% | 0.398 | - |
|  | PF3D7_1118800 | conserved Plasmodium protein, putative | 383/391 (98%) | 96.4% | 3.6% | 0.320 | 3 WD40 |
|  | PF3D7_1116400 | ER membrane protein Sec12 (SEC12) | 412/485 (85%) | 95% | 5.0% | 0.262 | 4 WD40 + 1 Transmembrane |
|  | PF3D7_1033500 | WD-repeat protein, putative | 602/709 (85%) | 90.5% | 9.5% | 0.308 | 4 WD40 |
|  | PF3D7_1348700 | conserved Plasmodium protein, Putative | 657/715 (92%) | 93.8% | 6.2% | 0.481 | 8 WD40 |
|  | PF3D7_1209400 | conserved Plasmodium protein, unknown function | 649/792 (82%) | 92.5% | 7.5% | 0.316 | 7 WD40 |
